# Prospective Associations of Daily Step Counts and Intensity With Cancer and Cardiovascular Disease Incidence and Mortality and All-Cause Mortality

**DOI:** 10.1001/jamainternmed.2022.4000

**Published:** 2022-09-12

**Authors:** Borja del Pozo Cruz, Matthew N. Ahmadi, I-Min Lee, Emmanuel Stamatakis

**Affiliations:** 1 Department of Sports Science and Clinical Biomechanics, Centre for Active and Healthy Ageing, University of Southern Denmark, Odense, Denmark; 2 Faculty of Medicine and Health, Charles Perkin Centre, School of Health Sciences, The University of Sydney, Camperdown, New South Wales, Australia; 3 Division of Preventive Medicine, Brigham & Women’s Hospital, Harvard Medical School, Boston, Massachusetts; 4 Department of Epidemiology, Harvard T. H. Chan School of Public Health, Boston, Massachusetts

## Abstract

**Importance:**

Recommendations for the number of steps per day may be easier to enact for some people than the current time- and intensity-based physical activity guidelines, but the evidence to support steps-based goals is limited.

**Objective:**

To describe the associations of step count and intensity with all-cause mortality and cancer and cardiovascular disease (CVD) incidence and mortality.

**Design, Setting, and Participants:**

This population-based prospective cohort study used data from the UK Biobank for 2013 to 2015 (median follow-up, 7 years) and included adults 40 to 79 years old in England, Scotland, and Wales. Participants were invited by email to partake in an accelerometer study. Registry-based morbidity and mortality were ascertained through October 2021. Data analyses were performed during March 2022.

**Exposures:**

Baseline wrist accelerometer-measured daily step count and established cadence-based step intensity measures (steps/min): incidental steps, (<40 steps/min), purposeful steps (≥40 steps/min); and peak-30 cadence (average steps/min for the 30 highest, but not necessarily consecutive, min/d).

**Main Outcomes and Measures:**

All-cause mortality and primary and secondary CVD or cancer mortality and incidence diagnosis. For cancer, analyses were restricted to a composite cancer outcome of 13 sites that have a known association with reduced physical activity. Cox restricted cubic spline regression models were used to assess the dose-response associations. The linear mean rate of change (MRC) in the log-relative hazard ratio for each outcome per 2000 daily step increments were also estimated.

**Results:**

The study population of 78 500 individuals (mean [SD] age, 61 [8] years; 43 418 [55%] females; 75 874 [97%] White individuals) was followed for a median of 7 years during which 1325 participants died of cancer and 664 of CVD (total deaths 2179). There were 10 245 incident CVD events and 2813 cancer incident events during the observation period. More daily steps were associated with a lower risk of all-cause (MRC, −0.08; 95% CI, −0.11 to −0.06), CVD (MRC, −0.10; 95% CI, −0.15 to −0.06), and cancer mortality (MRC, 95% CI, −0.11; −0.15 to −0.06) for up to approximately 10 000 steps. Similarly, accruing more daily steps was associated with lower incident disease. Peak-30 cadence was consistently associated with lower risks across all outcomes, beyond the benefit of total daily steps.

**Conclusions and Relevance:**

The findings of this population-based prospective cohort study of 78 500 individuals suggest that up to 10 000 steps per day may be associated with a lower risk of mortality and cancer and CVD incidence. Steps performed at a higher cadence may be associated with additional risk reduction, particularly for incident disease.

## Key Points

**Questions:**

What are the associations of daily step counts with cancer and cardiovascular disease incidence and mortality and all-cause mortality; and does the intensity of steps have additional benefits?

**Findings:**

This population-based prospective cohort study using UK Biobank data for 78 500 individuals (mean age, 61 years) found that more steps per day (up to about 10 000 steps) was associated with declines in mortality risks and decreased cancer and CVD incidence. Peak-30 cadence (stepping intensity) showed consistent associations with improved morbidity and mortality rates.

**Meaning:**

These findings indicate that accumulating more steps per day (up to about 10 000) may be associated with a lower risk of all-cause, cancer, and CVD mortality and incidence of cancer and CVD; higher step intensity may provide additional benefits.

## Teaser

This population-based prospective cohort study using UK Biobank data for 78 500 individuals with 7 years of follow-up examines associations of daily step counts and intensity with all-cause mortality and cancer and cardiovascular disease–related morbidity and mortality.

## Introduction

Evidence to date^1,2,3,4,5,6,7^ has prompted many to advocate for increasing daily steps as an important part of preventing chronic disease and premature mortality.^8,9,10,11^ Although this is popular advice, evidence to support the target of 10 000 steps per day for better health is scant. When Lee and colleagues^4^ found that as few as 4400 steps per day were associated with reduced mortality among older women,^12,13^ the finding led to debate and a call for more comprehensive evidence to inform step-based recommendations. Recent studies have suggested there is little additional risk reduction for all-cause mortality^5,7^ for more than 6000 to 8000 daily steps for those 60 years and older and 8000 to 10 000 steps for those younger than 60 years.

More steps have been associated with lower cardiovascular disease (CVD) mortality, but previous evidence is limited to older adults,^14,15^ high-risk populations,^1^ men only,^14^ women only,^16^ or cohorts with few mortality events,^17^ hindering the generalizability of the findings. More steps may lower the risks of cancer mortality, but evidence comes from only 1 study.^6^ Previous studies have missed the opportunity to explore beyond total daily steps to more specific step-based exposures (eg, differentiating between incidental vs purposeful steps). A more detailed analysis may be relevant for informing step-based recommendations.^18^ Moreover, the dose-response association between daily step counts and cancer incidence and CVD remain less explored.^9,10^ The intensity of the steps performed may also be relevant; however, current evidence is scarce, conflicting, and limited to mortality outcomes only.^3,4,6,19^ Besides, most evidence on step counts and intensity to date has relied on small cohort studies, which can make assessment of associations difficult, particularly for less common events.

This study examined the associations of daily step counts and step intensity with cancer and CVD incidence and all-cause, cancer-, and CVD-related mortality among a large sample of UK adults who wore wrist accelerometers.

## Methods

This prospective study was reviewed and approved by the National Health Service and the National Research Ethics Service (No. 11/NW/0382). Informed consent was waived because the study used deidentified publicly available data. The study followed the Strengthening the Reporting of Observational Studies in Epidemiology (STROBE) reporting guidelines.

### Study Design and Sample

This prospective study used data from the UK Biobank study.^20^ We included participants with valid accelerometer data and excluded those with poor self-rated health, prevalent cancer or CVD, and/or missing data for any of the covariates.

### Step Count and Intensity

From February 2013 through December 2015, a total of 236 462 individuals in the UK Biobank with a valid email address were sent an invitation to wear an accelerometer for 7 days. Of these, 103 684 individuals accepted the invitation and were mailed an Axivity AX3 accelerometer (Newcastle upon Tyne, UK) to wear on their dominant wrist for 24 hours per day for 7 days to measure physical activity. Compared with the larger UK Biobank population, participants who wore the accelerometer were generally healthier, higher socioeconomic status, and predominantly women (eTable 1 in the Supplement).

The AX3 accelerometers were initialized to collect data with a sampling frequency of 100 Hz and a dynamic range of ±8 g. Nonwear periods were identified according to standard procedures.^21,22^ Monitoring days were considered valid if wear time was greater than 16 hours.^21,22^ To be included, participants were required to have 3 or more valid monitoring days, including at least 1 weekend day, and to have worn the monitor during sleep periods.^23,24^ Physical activity type was classified with an accelerometer-based activity machine-learning scheme covering sedentary behavior, small utilitarian movements, walking, and running.^25^ We calculated steps during periods of ambulation using the Verisene step-count algorithm for wrist accelerometers.^26^ As shown with greater detail in eTable 2 of the Supplement, primary exposures were daily step counts, calculated as median number of steps per day across all valid days; established^4,27^ cadence-based stepping metrics reflective of the free-living stepping context (incidental steps, <40 steps/min; purposeful steps, ≥40 steps/min); and stepping intensity (peak-30 cadence defined as average steps/min for the 30 highest, but not necessarily consecutive, min/d). Secondary exposures were steps performed at light (<100 steps/min),^27^ moderate (100-129 steps/min),^27^ vigorous (≥130 steps/min),^27^ and moderate-to-vigorous (≥100 steps/min)^27^ intensity and walking steps (eTable 2 in the Supplement).

### Morbidity and Mortality Ascertainment

Participants were followed up from September 30 to October 31, 2021, with deaths obtained through linkage with the National Health Service (NHS) Digital of England and Wales or the NHS Central Register and National Records of Scotland. Inpatient hospitalization data were provided by either the Hospital Episode Statistics for England, the Patient Episode Database for Wales, or the Scottish Morbidity Record for Scotland. Cancer data linkage was obtained through national cancer registries. For England and Wales, cancer diagnosis data were provided by the Medical Research Information Service (NHS Information Center). For Scotland, cancer diagnosis data were provided by the Information Services Division. Included in the definition of CVD were fatal and nonfatal coronary heart disease, stroke, and heart failure (eTable 3 in the Supplement). We limited the analyses to a composite cancer outcome of 13 sites shown to be associated with low physical activity by a previous study^28^: bladder, breast, colon, endometrial, esophageal adenocarcinoma, gastric cardia, head and neck, kidney, liver, lung, myeloid leukemia, myeloma, and rectal (eTable 3 in the Supplement). The number of events (fatal and nonfatal) for each of these 13 cancer sites is shown in eTable 4 in the Supplement. We included primary and secondary cancer and CVD diagnoses. Censoring dates varied among resources (September-October 2021).

### Statistical Analysis

We described the sample by tertiles of average daily step count and peak-30 cadence. Dose-response associations were assessed using Cox restricted cubic spline models, with trimmed observations at 1% and 99% of the distribution. We prespecified knots placed at the 10th, 50th, and 90th percentiles of the exposure distribution.^29^ The functional form of associations was assessed by a Wald test, a linear or nonlinear likelihood ratio comparison, and graphical inspection of the adjusted martingale residuals of the linear model. Results were reported in log-relative hazard ratios and associated 95% CIs. We also estimated the linear mean rate of change (MRC) in the log-relative hazard ratio (95% CI) for morbidity and mortality outcomes for each 2000 daily step increments. To compare the magnitude of associations across cadence-based metrics, we reported the MRC for each 10% increment of each exposure’s distribution. Absolute mortality risk for each mortality outcome was estimated using Poisson regression, adjusting first for age and sex (model 1), then for model 1 plus race, education, socioeconomic status, smoking, alcohol, fruit and vegetable intake, and sleep (model 2); and then for medication and family history of cancer and/or CVD (model 3). For cause-specific outcomes, we used Fine and Grey models to account for competing risks^30^; proportional hazard assumptions were tested through visual examination of the Schoenfeld residuals.

Based on a priori defined directed acyclic graph (eFigure 1 in the Supplement), we adjusted our models with the following potential covariates: age (years), sex (male/female), race (White; yes/no), education (university degree; yes/no), socioeconomic status (Townsend Deprivation Index), smoking (never/previous/current smoker), alcohol use (never/previous/occasional/within guidelines/double guidelines/>double guidelines^31^), fruit and vegetable consumption (servings/d), family history of cancer and/or CVD (yes/no), medication use (cholesterol, insulin, hypertension; yes/no), accelerometer-measured sleep time (min), and number of days accelerometer was worn. For incidental steps, models were mutually adjusted for purposeful steps. For light intensity steps, models were mutually adjusted for moderate and vigorous intensity steps. For peak-30 cadence, models were adjusted for daily steps.

We tested the effect modification of the associations between daily steps and the outcomes by age (modeled as continuous covariate).^5,7^ We represented the interaction models as contour maps.

We performed 3 sensitivity analyses: (1) models censoring participants when the event of interest was observed within the first 2 years of follow-up; (2) a model with further adjustment for body mass index (BMI; calculated as weight in kilograms divided by height in meters squared) where accelerometry-based sleep time and fruit and vegetable consumption were replaced with self-reported sleep ^32,33^ and dietary scores,^34,35^ respectively; and (3) additional model limiting the sample to participants who wore the accelerometer for 20 or more hours on each valid day. Statistical tests were 2-tailed and α levels of .05 were considered statistically significant. Data analyses were performed during March 2022 using R, version 4.2.1 (The R Foundation for Statistical Computing).

## Results

The sample for mortality outcomes included 78 500 participants (mean [SD] age, 61 [8] years; 43 418 [55%] female; 75 874 [97%] White and 2626 [3%] non-White individuals) and a median follow-up of 7.0 years (53 196 person-years). Of these, a total of 2179 participants died (of cancer, 1325; of CVD, 664) during the follow-up period. There were 10 245 incident CVD events during the median follow-up of 6.8 years (498 570 person-years). Corresponding figures for incident cancer were 2813 events and a median follow-up of 6.9 years (523 999 person-years; eFigure 2 in the Supplement).

Table 1 shows the baseline sample characteristics stratified by tertiles of daily steps. Participants who took more steps had lower BMI, experienced better sleep, and did not smoke or drink alcohol. Participants with higher peak-30 cadence were healthier, younger, and took more steps (Table 2). Participants with accelerometry excluded from the analysis were mostly female, less wealthy, non-White race, and less active than those included in the final analysis (eTable 5 in the Supplement).

**Table 1.** Baseline Characteristics of Study Participants by Tercile Category of Daily Accelerometer-Measured Steps^a^

Characteristic	Overall	Tercile 1 (1539 to <5385 steps)	Tercile 2 (5385 to <8821 steps)	Tercile 3 (≥8821 steps)
Participants, No.	78 500	26 167	26 164	26 169
Age (SD), y	61.1 (7.9)	62.9 (7.7)	60.9 (7.8)	59.6 (7.8)
Female, No. (%)	43 418 (55.3)	14 603 (55.8)	14 227 (54.4)	14 588 (55.7)
Male, No. (%)	35 082 (44.7)	11 563 (44.2)	11 938 (45.6)	11 581 (44.3)
Race, non-White, No. (%)	2626 (3.3)	936 (3.6)	862 (3.3)	828 (3.2)
University education, No. (%)	43 404 (55.3)	14 815 (56.6)	14 269 (54.5)	14 320 (54.7)
Townsend Deprivation Index^b^	−1.77 (2.8)	−1.72 (2.8)	−1.80 (2.79)	−1.79 (2.8)
Smoking, No. (%)				
Never	45 361 (57.8)	14 620 (55.9)	15 222 (58.2)	15 519 (59.3)
Previous	27 884 (35.5)	9535 (36.4)	9217 (35.2)	9132 (34.9)
Current	5255 (6.7)	2012 (7.7)	1725 (6.6)	1518 (5.8)
Alcohol use, No. (%)^c^				
Never	2181 (2.8)	823 (3.1)	664 (2.5)	694 (2.7)
Previous	1990 (2.5)	718 (2.7)	629 (2.4)	643 (2.5)
Occasional	15 683 (20.0)	5824 (22.3)	5014 (19.2)	4845 (18.5)
Within guidelines	28 933 (36.9)	9334 (35.7)	9774 (37.4)	9825 (37.5)
Double guidelines	18 655 (23.8)	5847 (22.3)	6336 (24.2)	6472 (24.7)
>Double guidelines	11 058 (14.1)	3621 (13.8)	3747 (14.3)	3690 (14.1)
Fruit servings/d	3.22 (2.5)	3.09 (2.5)	3.19 (2.4)	3.37 (2.6)
Vegetable servings/d	4.89 (3.1)	4.81 (3.0)	4.89 (3.2)	4.98 (3.1)
Family history of CVD, No. (%)	42 922 (54.7)	14 822 (56.6)	14 245 (54.4)	13 855 (52.9)
Family history of cancer, No. (%)	19 568 (24.9)	6681 (25.5)	6554 (25.0)	6333 (24.2)
Cholesterol medication, No. (%)	10 664 (13.6)	4864 (18.6)	3302 (12.6)	2498 (9.5)
Insulin medication, No. (%)	472 (0.6)	225 (0.9)	138 (0.5)	109 (0.4)
Hypertension medication, No. (%)	12 504 (15.9)	5598 (21.4)	3915 (15.0)	2991 (11.4)
Sleep (accelerometer-measured, min/d)	421.55 (88.6)	416.5 (96.0)	423.2 (86.9)	424.9 (82.0)
Accelerometer worn, d	6.90 (0.4)	6.91 (0.4)	6.9 (0.4)	6.9 (0.4)
Steps/d^d^				
Total	7198.2 (4609.2)	3237.6 (960.9)	6181.7 (898.7)	12 174.8 (4529.8)
Incidental	3240.6 (1272.4)	2082.4 (609.2)	3235.7 (730.8)	4403.5 (1121.7)
Purposeful	4621.8 (4159.4)	1619.0 (1056.8)	3610.7 (1457.4)	8635.4 (4744.7)
LIPA	6553.3 (3399.5)	3464.6 (1054.9)	6010.8 (1136.3)	10 184.2 (3041.3)
MPA	614.9 (757.8)	185.1 (192.8)	441.0 (318.2)	1218.5 (1002.4)
VPA	870.8 (1810.1)	244.07 (609.8)	578.3 (969.8)	1789.9 (2682.2)
MVPA	1485.7 (2323.5)	429.2 (709.4)	1019.3 (1132.5)	3008.4 (3279.9)
Peak 30-min cadence	75.71 (31.5)	48.63 (12.7)	73.3 (14.4)	105.2 (31.6)

Abbreviations: CVD, cardiovascular disease; LIPA, light intensity physical activity; MPA, moderate intensity physical activity; MVPA, moderate-to-vigorous physical activity; VPA, vigorous intensity physical activity.

^a^ Unless otherwise noted, data are reported as mean (SD) values.

^b^ Lower score indicates more affluence.

^c^ Guidelines for alcohol use in the UK recommend no more than 14 units (1 unit = 10 mL of pure alcohol) per week for both men and women.

^d^ Total steps/d, calculated as median number of steps per day across all valid days; incidental steps, total steps/d at 1-39 steps/min; purposeful steps, total steps/d at ≥40 steps/min; LIPA steps/d, total steps/d at <100 steps/min; MPA steps/d, total steps/d at ≥100 and <130 steps/min; MVPA steps/d, total steps/d at ≥130 steps/min; MVPA steps/d, total steps/d at ≥100 steps/min; peak 30-min cadence, average steps/min recorded for the 30 highest (but not necessarily consecutive) min/d.

**Table 2.** Baseline Characteristics of Study Participants by Tercile Category of Peak 30-Min Cadence^a^

Characteristic	Overall	Tercile 1 (0 to <60 steps)	Tercile 2 (60 to <83 steps)	Tercile 3 (≥83 steps)
Participants, No.	78 500	26 167	26 148	26 185
Age (SD), y	61.1 (7.9)	62.9 (7.6)	60.9 (7.8)	59.5 (7.8)
Female, No. (%)	43 418 (55.3)	14 997 (57.3)	14 450 (55.3)	13 971 (53.4)
Male, No. (%)	35 082 (44.7)	11 170 (42.7)	11 698 (44.7)	12 214 (46.6)
Race, non-White, No. (%)	2626 (3.3)	861 (3.3)	867 (3.3)	898 (3.4)
University education, No. (%)	43 404 (55.3)	14 942 (57.1)	14 560 (55.7)	13 902 (53.1)
Townsend Deprivation Index^b^	−1.8 (2.8)	−1.8 (2.8)	−1.9 (2.8)	−1.7 (2.8)
Smoking, No. (%)				
Never	45 361 (57.8)	14 527 (55.5)	15 193 (58.1)	15 641 (59.7)
Previous	27 884 (35.5)	9613 (36.7)	9290 (35.5)	8981 (34.3)
Current	5255 (6.7)	2027 (7.7)	1665 (6.4)	1563 (6.0)
Alcohol use, No. (%)^c^				
Never	2181 (2.8)	829 (3.2)	660 (2.5)	692 (2.6)
Previous	1990 (2.5)	707 (2.7)	651 (2.5)	632 (2.4)
Occasional	15 683 (20.0)	5829 (22.3)	5035 (19.3)	4819 (18.4)
Within guidelines	28 933 (36.9)	9373 (35.8)	9757 (37.3)	9803 (37.4)
Double guidelines	18 655 (23.8)	5854 (22.4)	6345 (24.3)	6456 (24.7)
>Double guidelines	11 058 (14.1)	3575 (13.7)	3700 (14.2)	3783 (14.4)
Fruit servings/d	3.22 (2.5)	3.11 (2.4)	3.20 (2.4)	3.3 (2.6)
Vegetable servings/d	4.89 (3.1)	4.9 (3.1)	4.9 (3.1)	4.9 (3.2)
Family history of CVD, No. (%)	42 922 (54.7)	14 906 (57.0)	14251 (54.5)	13 765 (52.6)
Family history of cancer, No. (%)	19 568 (24.9)	6748 (25.8)	6554 (25.1)	6266 (23.9)
Cholesterol medication, No. (%)	10 664 (13.6)	4846 (18.5)	3322 (12.7)	2496 (9.5)
Insulin medication, No. (%)	472 (0.6)	230 (0.9)	136 (0.5)	106 (0.4)
Hypertension medication, No. (%)	12 504 (15.9)	5635 (21.5)	3939 (15.1)	2930 (11.2)
Sleep (accelerometer-measured, min/d)	421.55 (88.56)	414.51 (95.35)	422.76 (87.49)	427.37 (81.85)
Accelerometer worn, d	6.90 (0.37)	6.91 (0.37)	6.90 (0.37)	6.90 (0.39)
Steps/d^d^				
Total	7198.2 (4609.2)	3471.8 (1285.9)	6500.9 (1837.7)	11 618.3 (4976.6)
Incidental	3240.6 (1272.4)	2349.8 (866.8)	3445.6 (1095.4)	3925.9 (1265.1)
Purposeful	4621.8 (4159.36)	1522.1 (903.06)	3689.3 (1358.10)	8650.6 (4744.16)
LIPA	6553.3 (3399.5)	3729.9 (1346.1)	6455.0 (1907.5)	9472.8 (3564.2)
MPA	614.9 (757.8)	144.4 (137.2)	400.8 (217.9)	1298.8 (959.4)
VPA	870.8 (1810.1)	178.7 (493.8)	461.4 (808.8)	1971.2 (2658.8)
MVPA	1485.7 (2323.5)	323.1 (559.6)	862.2 (897.4)	3269.9 (3185.2)
Peak 30-min cadence	75.71 (31.5)	46.59 (9.9)	71.34 (6.5)	109.2 (29.2)

Abbreviations: CVD, cardiovascular disease; LIPA, light intensity physical activity; MPA, moderate intensity physical activity; MVPA, moderate-to-vigorous physical activity; VPA, vigorous intensity physical activity.

^a^ Unless otherwise noted, data are reported as mean (SD) values.

^b^ Lower score indicates more affluence.

^c^ Guidelines for alcohol use in the UK recommend no more than 14 units (1 unit = 10 mL of pure alcohol) per week for both men and women.

^d^ Total steps/d, calculated as median number of steps per day across all valid days; incidental steps, total steps/d at 1-39 steps/min; purposeful steps, total steps/d at ≥40 steps/min; LIPA steps/d, total steps/d at <100 steps/min; MPA steps/d, total steps/d at ≥100 and <130 steps/min; MVPA steps/d, total steps/d at ≥130 steps/min; MVPA steps/d, total steps/d at ≥100 steps/min; peak 30-min cadence, average steps/min recorded for the 30 highest (but not necessarily consecutive) min/d.

### All-Cause, Cancer, and CVD Mortality

**Figure 1.**
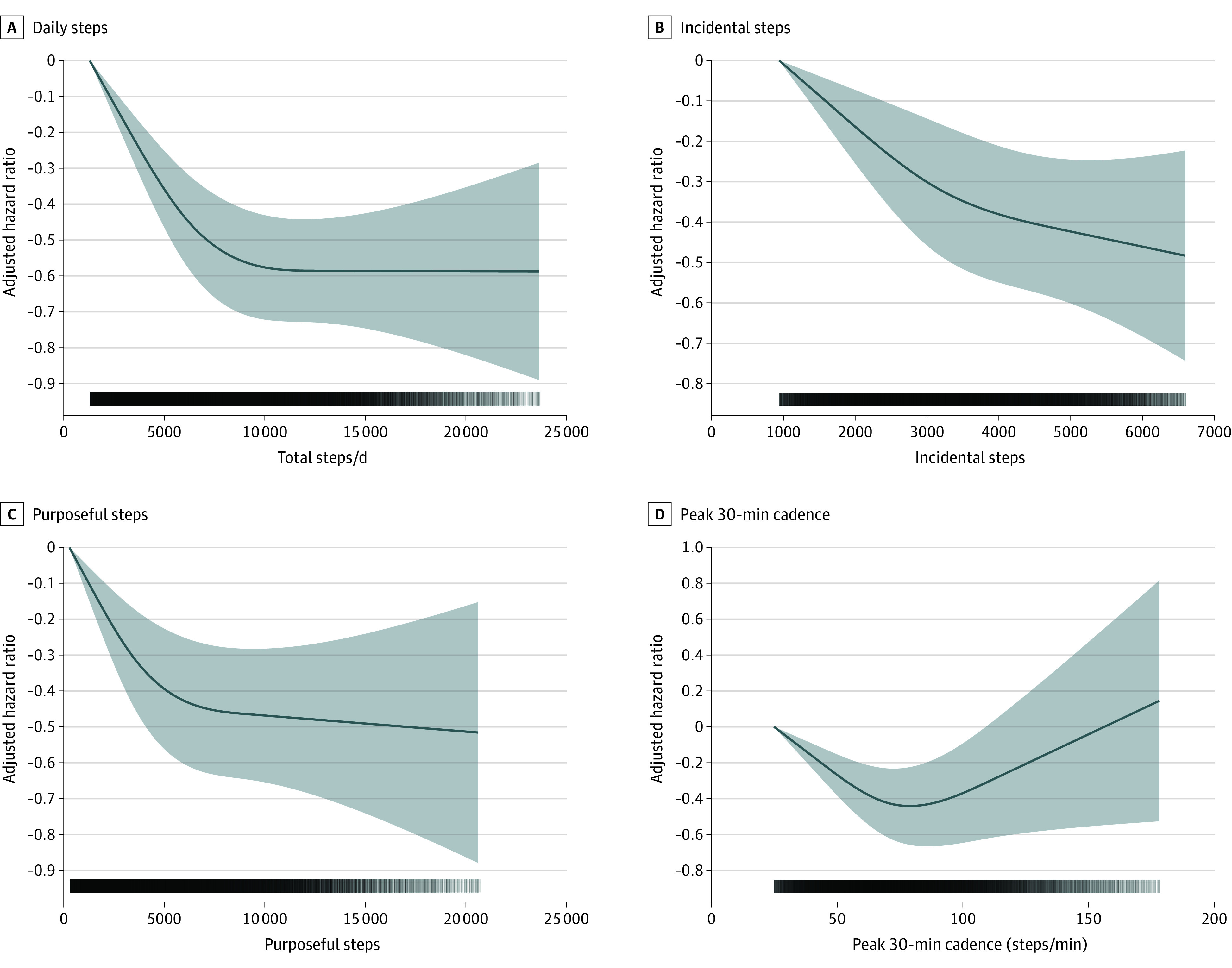
Dose-Response Associations Between Primary Exposures and All-Cause Mortality Hazard ratios and associated 95% CIs adjusted for age, sex, race, education, Townsend Deprivation Index, smoking, alcohol use, fruit and vegetable consumption, family history of cancer and CVD, medication use (cholesterol, insulin, hypertension), accelerometer-measured sleep, and wear accelerometer days. For incidental steps, models were further adjusted for purposeful steps (and vice versa). Dose-response associations were assessed with restricted cubic splines with knots at 10th, 50th, and 90th percentiles of the distribution of the exposure of interest. Darker colors in the lower bars represent a higher sample clustering. Shaded areas represent 95% CIs. CVD indicates cardiovascular disease; total steps/d, median number of steps per day across valid days; incidental steps, total daily steps at 1-39 steps/min; purposeful steps, total daily steps at ≥40 steps/min; peak 30-min cadence, average steps/min recorded for the 30 highest, but not necessarily consecutive, min/day.

**Figure 2.**
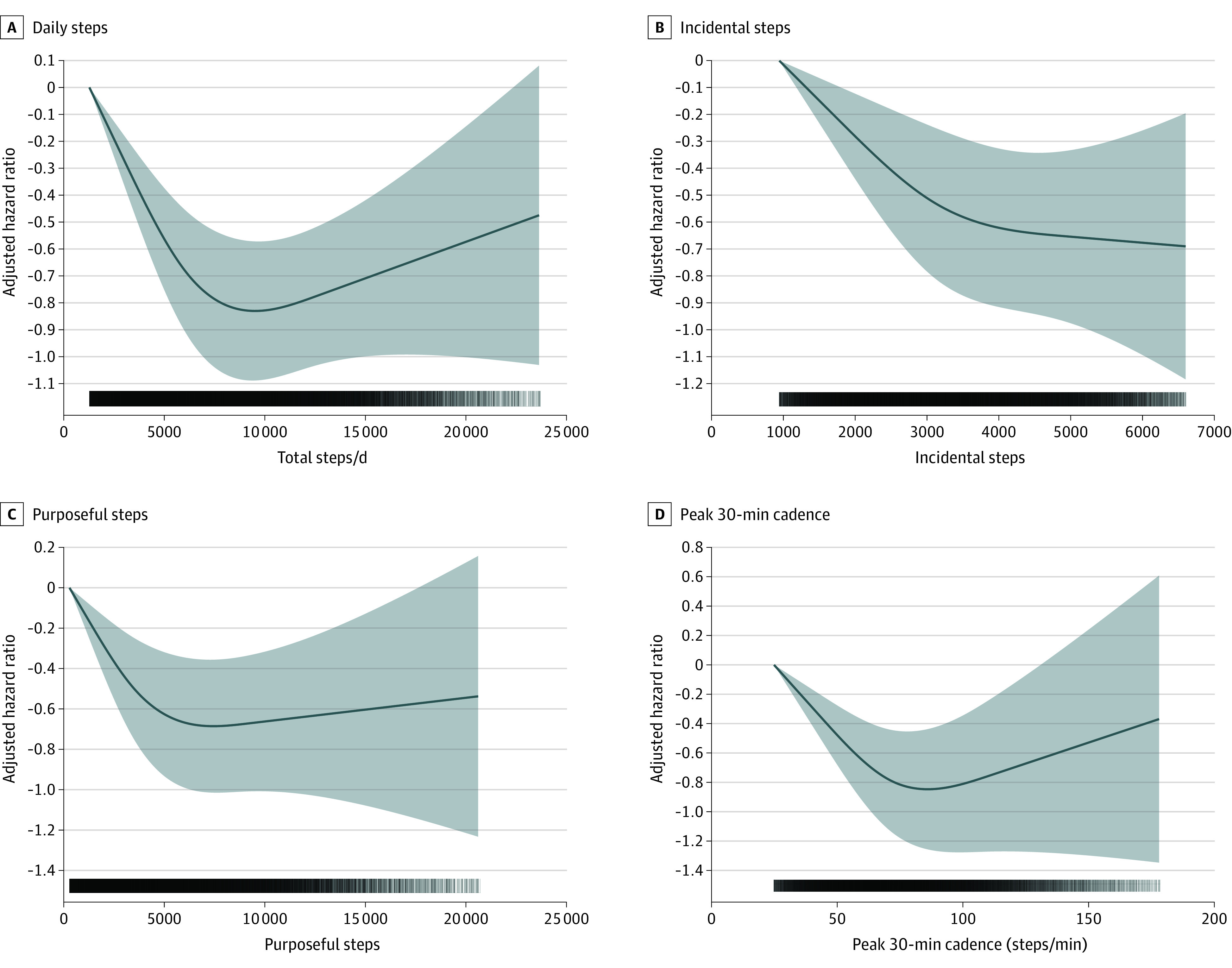
Dose-Response Association Between Primary Exposures and CVD Mortality Hazard ratios and associated 95% CIs adjusted for age, sex, race, education, Townsend Deprivation Index, smoking, alcohol use, fruit and vegetable consumption, family history of cancer and CVD, medication use (cholesterol, insulin, hypertension), accelerometer-measured sleep, and wear accelerometer days. For incidental steps, models were further adjusted for purposeful steps (and vice versa). Dose-response associations were assessed with restricted cubic splines with knots at 10th, 50th, and 90th percentiles of the distribution of the exposure of interest. Darker colors in the lower bars represent a higher sample clustering. Shaded areas represent 95% CIs. CVD indicates cardiovascular disease; total steps/d, median number of steps per day across valid days; incidental steps, total daily steps at 1-39 steps/min; purposeful steps, total daily steps at ≥40 steps/min; peak 30-min cadence, average steps/min recorded for the 30 highest, but not necessarily consecutive, min/day.

**Figure 3.**
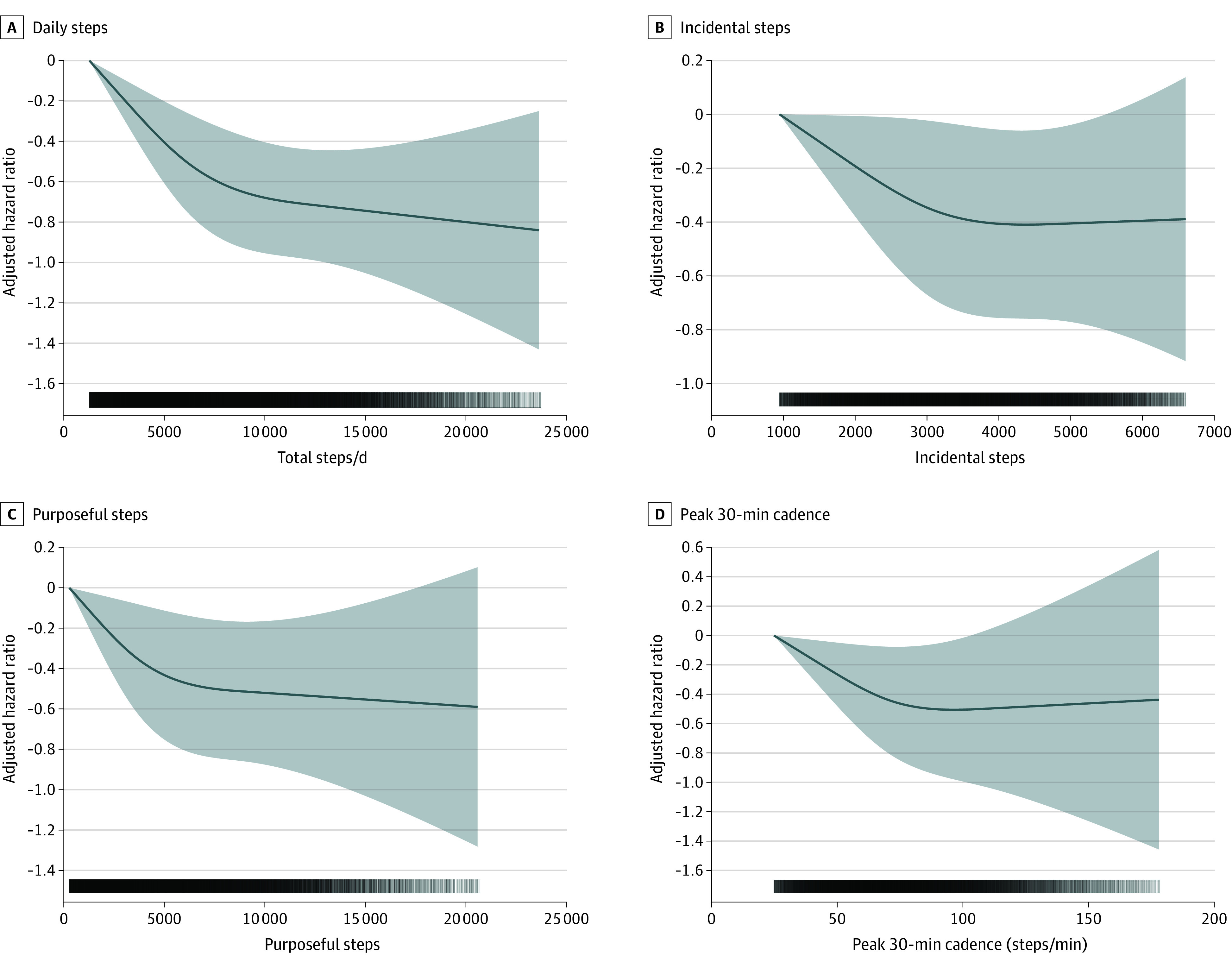
Dose-Response Association Between Primary Exposures and Cancer Composite of 13 Sites With a Known Relationship With Low Physical Activity^28^ Hazard ratios and associated 95% CIs adjusted for age, sex, race, education, Townsend Deprivation Index, smoking, alcohol use, fruit and vegetable consumption, family history of cancer and CVD, medication use (cholesterol, insulin, hypertension), accelerometer-measured sleep, and wear accelerometer days. For incidental steps, models were further adjusted for purposeful steps (and vice versa). Dose-response associations were assessed with restricted cubic splines with knots at 10th, 50th, and 90th percentiles of the distribution of the exposure of interest. Darker colors in the lower bars represent a higher sample clustering. Shaded areas represent 95% CIs. CVD indicates cardiovascular disease; total steps/d, median number of steps per day across valid days; incidental steps, total daily steps at 1-39 steps/min; purposeful steps, total daily steps at ≥40 steps/min; peak 30-min cadence, average steps/min recorded for the 30 highest, but not necessarily consecutive, min/day.

We found no evidence of violation of the proportional hazard assumption (eFigure 3 in the Supplement). Higher number of daily steps was associated with a lower risk of all-cause (MRC for each 2000 steps increment, 0.08; 95% CI, −0.11 to −0.06), cancer (MRC for each 2000 steps increment, 0.11; 95% CI, 0.15 to −0.06) and CVD (MRC for each 2000 steps increment, −0.10; 95% CI, 0.15 to −0.06), and mortality (Figure 1). A higher number of incidental steps was associated with a lower risk of all-cause (MRC for each 10% incidental steps increment [+10%], −0.06; 95% CI, −0.05 to −0.07), cancer (MRC +10% incidental, −0.06; 95% CI, −0.8 to −0.04), and CVD (MRC +10% incidental, −0.10; 95% CI, −0.11 to −0.09) mortality. A higher number of purposeful steps was associated with a lower risk of mortality for all-cause (MRC +10% purposeful, −0.07; 95% CI, −0.10 to −0.03), cancer (MRC +10% purposeful, −0.08; 95% CI, −0.15 to −0.02) and CVD (MRC +10% purposeful, −0.10; 95% CI, −0.17 to −0.02) mortality. Consistent associations of a stronger magnitude were found for peak-30 cadences, beyond the benefits of total daily steps for all-cause (MRC +10% 30-min cadence, −0.08; 95% CI, −0.10 to −0.05), cancer (MRC +10% 30-min cadence, −0.09; 95% CI, −0.13 to −0.05), and CVD (MRC +10% 30-min cadence, −0.14 ; 95% CI, −0.18 to −0.10) mortality (Figures 2 and 3). For the secondary exposures, the associations were similar with the exception of the analyses of vigorous intensity stepping and cancer and CVD mortality (eFigures 4-6 in the Supplement). Absolute risks for mortality outcomes are shown in eFigures 7-10 in the Supplement. The absolute all-cause mortality rate reduction comparing extreme quintiles was 8.8 and 8.1 deaths per 10 000 person-years for daily steps peak-30 cadence, respectively.

### Cancer and CVD Incidence

Increasing numbers of daily steps (MRC for each 2000 steps increment, −0.04; 95% CI, −0.03 to −0.06), purposeful steps (MRC for +10% purposeful, −0.04; 95% CI, −0.05 to −0.02), and peak-30 cadence (MRC +10% peak-30 cadence increment, −0.07; 95% CI, −0.08 to −0.06) were associated with lower CVD incidence (eFigure 11 in the Supplement). Similar patterns were observed for cancer incidence (Figure 3 and eFigure 12 in the Supplement). Higher number of walking steps and light intensity steps were associated with a lower risk of cancer and CVD incidence (eFigures 13 and 14 in the Supplement). We found additional associations between moderate and moderate-to-vigorous intensity steps and risk of incident cancer (eFigure 14 in the Supplement).

### Additional and Sensitivity Analyses

There was no evidence of effect modification by age on the associations between daily step count and the outcomes examined (eFigure 15 in the Supplement). Censoring participants when the event of interested occurred within the first 2 years of follow-up mirrored the results of the main analysis (eFigures 16-20 in the Supplement). Adjusting for BMI, self-reported sleep characteristics, and dietary pattern (eFigure 21-25 in the Supplement) or restricting the sample to participants who wore the accelerometer for 20 or more hours per valid day (eFigure 26-30 in the Supplement) did not change the patterns of association, although the magnitude of associations was generally attenuated.

## Discussion

This large-scale accelerometry study of 78 500 adults from age 40 to 79 years contributes additional data on the associations of daily amounts and intensity of walking with mortality and incident disease. These findings are relevant for public health. We found no minimal threshold for the association of daily steps with mortality and morbidity. These associations were observed for up to approximately 10 000 steps per day, a threshold above which the level of statistical uncertainty may have blurred the true dose-response relationship. Similar patterns were observed for cancer and CVD incidence. Incidental steps were also associated with lower risk of mortality and morbidity. Nevertheless, purposeful steps and particularly peak-30 cadence were consistently associated with lower risk of all-cause mortality and cancer and CVD morbidity and mortality in this study.

### All-Cause, Cancer, and CVD Mortality

We found evidence of an inverse dose-response association between daily steps and all-cause, cancer, and CVD mortality up to approximately 10 000 steps per day, a threshold that is 20% higher than previously observed in participants age 60 years and older but similar to that seen in younger participants.^5,7^ Most existing consumer-grade activity trackers are wrist-worn, and our data may represent more relevant targets for the population compared with previous studies.^36^ These study results highlight the potential value of higher amounts of daily steps (ie, ~10 000 steps per day; absolute risk reduction, 36% vs baseline level) for optimal health; however, the proportion of the population currently achieving this goal is low (~20% in this sample). We did not find a minimal threshold for the beneficial association between stepping and mortality risk in this sample. Other studies have reported similar findings.^4^ Promotion of lower step targets may provide a more realistic and achievable goal for the general adult population where longevity gains may be maximized simply by shifting away from the least-active end of the step-count distribution. Peak-30 cadence was consistently associated with lower mortality risks (absolute risk reduction difference between extreme quintiles, 34%), a finding that reflects the importance of the *natural best effort* relative to the individual’s capability and may better represent the amount of activity at higher intensities than other metrics.

### Cancer and CVD Incidence

Previous cross-sectional evidence has shown a correlation between increased walking and a lower risk of CVD prevalence.^37^ We provide novel evidence of a prospective association of step counts with CVD mortality and incidence. Previous randomized clinical trials^38^ have shown the robust effects of walking on several CVD risk factors. We identified similar data patterns for cancer incidence. Notably, we detected an association between incidental steps and a lower risk of both cancer and CVD incidence. This finding warrants further attention because incidental walking throughout the day may be more feasible for some people and may be associated with additional health benefits, more so than purposeful steps. Similarly, peak-30 cadence was inversely and consistently associated with cancer and CVD incidence. Our study contributes critical evidence toward step count−based recommendations, which could be particularly easy to communicate, interpret, and measure.^4,9,10^ Step counts may be especially relevant for people who mostly perform incidental, unstructured, and unplanned physical activity. For individuals who are not intentionally tracking their physical activity, it may be challenging to recall time-based physical activity amounts or determine whether they are sufficiently active in relation to the current minute- and intensity-based guidelines.^11^ Therefore, step-based guidelines could provide useful supplementary recommendations to the current physical activity guidelines.

### Strengths and Limitations

This study had several strengths. First, to our knowledge, this was the largest population sample and analysis of adults using accelerometry and linkage to prospective outcomes. The device and protocol used allowed us to collect 24 h of accelerometry data to reduce the chance of missing periods of ambulatory activity. We used registry-based prospectively collected data, which increased the internal validity of our estimates. Unlike previous studies,^3,4^ we accounted for the presence of competing risks for event-specific outcomes. We also took measures to minimize the likelihood of reverse causation by censoring all the events that occurred during the first 2 years of follow-up. Lastly, the machine learning algorithm we used allowed us to separate walking steps from other ambulatory activity, thus overcoming some common limitations of previous studies.^3,4,19^

This study also had limitations. First, the observational design of this study precludes us from making causal claims. Second, the step-count data were collected only once at baseline and may not be representative of habitual walking behavior. Nevertheless, we found consistent daily steps in participants with available repeated accelerometer wear approximately 4 years later (n = 3400; intraclass correlation, 0.76). Third, despite censoring participants who had the event of interest within the first 2 years of follow-up, some potential for reverse causation may still exist. Fourth, although a priori defined directed acyclic graph was used to identify key factors known to influence the causal associations between steps and mortality and disease incidence outcomes, residual or unmeasured confounding may still be present. Fifth, covariates were not measured at accelerometer wear date. Nonetheless, responses to the selected covariates were relatively stable over time, and therefore, the associations between accelerometer-assessed physical activity and health outcomes are valid.^39^ Sixth, the UK Biobank had a very low response rate (5.5% of those invited; 45% of UK Biobank participants for the accelerometry study; mortality rate, 6.1 and 4.1 per 1000 person-years, respectively) and participants were not representative of the overall UK population.^40^ However, recent studies have demonstrated that the lack of representativeness in the UK Biobank does not affect the associations of physical activity with disease incidence and mortality outcomes.^41,42^ The uptick of the right part of the dose-response curves in this study likely reflects the sparsity of data and/or events rather than a genuine lack of beneficial association at higher levels of stepping. This is particularly evident after approximately 10 000 steps per day, which may represent the health consciousness of the participants in the study around this target. Finally, because the relative energy cost of walking and other daily activities is higher in older adults than younger adults, the observed benefits of daily step counts may vary depending on the interaction between step intensity and age. Hence, a single recommendation for step count may not be appropriate for all adults; however, this interaction was not observed in our sample.

## Conclusions

This population-based prospective cohort study using UK Biobank data for 78 500 individuals suggests that accruing more steps per day (up to ~10 000 steps/d) was associated with steady declines in mortality risks, beyond which the associations were less evident. There was no minimal threshold for the beneficial association of increasing the number of daily steps with mortality and morbidity. If combined with effective behavioral strategies, this information could be used to motivate the least active individuals to increase their steps and the more active individuals to reach the 10 000-step target. Daily steps were also associated with cancer and CVD incidence. Peak-30 cadence was consistently associated with morbidity and mortality. These findings can inform future evidence-based physical activity recommendations using daily steps.

## Notes

Supplement. eTable 1. Baseline characteristics of participants who wore the accelerometer and those who did not in the UK Biobank eTable 2. Stepping-based exposure variables definition eTable 3. Assessment of CVD and cancer incidence (fatal and non-fatal) and definition of diseases eTable 4. Tabulation of the number of events per physical activity-related cancer site considered in this study eTable 5. Characteristics of accelerometer sample excluded from analysis eFigure 1. A priori Defined Directed Acyclic Graph eFigure 2. Flow diagram of participants in the study eFigure 3. Adjusted Schoenfeld residuals (daily step counts) eFigure 4. Dose–response association (Adjusted hazard ratios and associated 95% confidence interval band) between secondary exposures and all-cause mortality. eFigure 5. Dose–response association (Adjusted hazard ratios and associated 95% confidence interval band) between secondary exposures and CVD mortality. eFigure 6. Dose–response association (Adjusted hazard ratios and associated 95% confidence interval band) between secondary exposures and physical activity-related cancer composite 13 sites mortality outcome. eFigure 7. Sequentially adjusted dose–response quintile-based absolute risk describing the associations between daily steps and mortality outcomes (events rate per 10000 person-years and associated 95% confidence intervals) eFigure 8. Sequentially adjusted dose–response quintile-based absolute risk describing the associations between incidental steps and mortality outcomes (events rate per 10000 person-years and associated 95% confidence intervals) eFigure 9. Sequentially adjusted dose–response quintile-based absolute risk describing the associations between purposeful steps and mortality outcomes (events rate per 10000 person-years and associated 95% confidence intervals). eFigure 10. Sequentially adjusted dose–response quintile-based absolute risk describing the associations between peak-30 cadence and mortality outcomes (events rate per 10000 person-years and associated 95% confidence intervals) eFigure 11. Dose–response association (Adjusted^a^ hazard ratios and associated 95% confidence interval band) between primary exposures and CVD incidence. eFigure 12. Dose-response association (Adjusted^a^ hazard ratios and associated 95% confidence interval band) between primary exposures and physical activity-related cancer composite 13 sites incidence outcome. eFigure 13. Dose–response association (Adjusted^a^ hazard ratios and associated 95% confidence interval band) between secondary exposures and CVD incidence. eFigure 14. Dose–response association (Adjusted^a^ hazard ratios and associated 95% confidence interval band) between secondary exposures and physical activity-related cancer composite 13 sites incidence outcome. eFigure 15. Age modification effects eFigure 16. Dose–response association (Adjusted^a^ hazard ratios and associated 95% confidence interval band) between primary exposures and all-cause mortality (Sensitivity analysis: left-truncation at 2 years follow-up). eFigure 17. Dose–response association (Adjusted^a^ hazard ratios and associated 95% confidence interval band) between primary exposures and CVD mortality (Sensitivity analysis: left-truncation at 2 years follow-up). eFigure 18. Dose–response association (Adjusted^a^ hazard ratios and associated 95% confidence interval band) between primary exposures and physical activity-related cancer composite 13 sites mortality outcome (Sensitivity analysis: left-truncation at 2 years follow-up). eFigure 19. Dose–response association (Adjusted^a^ hazard ratios and associated 95% confidence interval band) between primary exposures and CVD incidence (Sensitivity analysis: left-truncation at 2 years follow-up). eFigure 20. Dose–response association (Adjusted^a^ hazard ratios and associated 95% confidence interval band) between primary exposures and physical activity-related cancer composite 13 sites incidence outcome (Sensitivity analysis: left-truncation at 2 years follow-up). eFigure 21. Dose–response association (Adjusted^a^ hazard ratios and associated 95% confidence interval band) between primary exposures and all-cause mortality (Sensitivity analysis: addition of body mass index, and sleep and dietary score). eFigure 22. Dose–response association (Adjusted^a^ hazard ratios and associated 95% confidence interval band) between primary exposures and CVD mortality (Sensitivity analysis: addition of body mass index, and sleep and dietary score). eFigure 23. Dose–response association (Adjusted^a^ hazard ratios and associated 95% confidence interval band) between primary exposures and physical activity-related cancer composite 13 sites mortality outcome (Sensitivity analysis: addition of body mass index, and sleep and dietary score). eFigure 24. Dose–response association (Adjusted^a^ hazard ratios and associated 95% confidence interval band) between primary exposures and CVD incidence (Sensitivity analysis: addition of body mass index, and sleep and dietary score). eFigure 25. Dose–response association (Adjusted^a^ hazard ratios and associated 95% confidence interval band) between primary exposures and physical activity-related cancer composite 13 sites incidence outcome (Sensitivity analysis: addition of body mass index, and sleep and dietary score). eFigure 26. Dose–response association (Adjusted^a^ hazard ratios and associated 95% confidence interval band) between primary exposures and all-cause mortality (Sensitivity analysis: restricted to participants with valid wear time days of 20h or more). eFigure 27. Dose–response association (Adjusted^a^ hazard ratios and associated 95% confidence interval band) between primary exposures and CVD mortality (Sensitivity analysis: restricted to participants with valid wear time days of 20h or more). eFigure 28. Dose–response association (Adjusted^a^ hazard ratios and associated 95% confidence interval band) between primary exposures and physical activity-related cancer composite 13 sites mortality outcome (Sensitivity analysis: restricted to participants with valid wear time days of 20h or more). eFigure 29. Dose–response association (Adjusted^a^ hazard ratios and associated 95% confidence interval band) between primary exposures and CVD incidence (Sensitivity analysis: restricted to participants with valid wear time days of 20h or more). eFigure 30. Dose–response association (Adjusted^a^ hazard ratios and associated 95% confidence interval band) between primary exposures and physical activity-related cancer composite 13 sites incidence outcome (Sensitivity analysis: restricted to participants with valid wear time days of 20h or more).
