# Supplementary material for: Prospective Associations of Daily Step Counts and Intensity With Cancer and Cardiovascular Disease Incidence and Mortality and All-Cause Mortality
Source: JAMA Intern Med. 2022 Sep 12;182(11):1139–48. doi: 10.1001/jamainternmed.2022.4000 (PMC9468953; doi:10.1001/jamainternmed.2022.4000)
Supplement: Supplement. — eTable 1. Baseline characteristics of participants who wore the accelerometer and those who did not in the UK Biobank eTable 2. Stepping-based exposure variables definition eTable 3. Assessment of CVD and cancer incidence (fatal and non-fatal) and definition of diseases eTable 4. Tabulation of the number of events per physical activity-related cancer site considered in this study eTable 5. Characteristics of accelerometer sample excluded from analysis eFigure 1. A priori Defined Directed Acyclic Graph eFigure 2. Flow diagram of participants in the study eFigure 3. Adjusted Schoenfeld residuals (daily step counts) eFigure 4. Dose–response association (Adjusted hazard ratios and associated 95% confidence interval band) between secondary exposures and all-cause mortality. eFigure 5. Dose–response association (Adjusted hazard ratios and associated 95% confidence interval band) between secondary exposures and CVD mortality. eFigure 6. Dose–response association (Adjusted hazard ratios and associated 95% confidence interval band) between secondary exposures and physical activity-related cancer composite 13 sites mortality outcome. eFigure 7. Sequentially adjusted dose–response quintile-based absolute risk describing the associations between daily steps and mortality outcomes (events rate per 10000 person-years and associated 95% confidence intervals) eFigure 8. Sequentially adjusted dose–response quintile-based absolute risk describing the associations between incidental steps and mortality outcomes (events rate per 10000 person-years and associated 95% confidence intervals) eFigure 9. Sequentially adjusted dose–response quintile-based absolute risk describing the associations between purposeful steps and mortality outcomes (events rate per 10000 person-years and associated 95% confidence intervals). eFigure 10. Sequentially adjusted dose–response quintile-based absolute risk describing the associations between peak-30 cadence and mortality outcomes (events rate per 10000 [file jamainternmed-e224000-s001.pdf]

Supplemental Online Content

del Pozo Cruz B, Ahmadi MN, Lee IM, Stamatakis E. Prospective associations of daily step counts and intensity with cancer and cardiovascular disease incidence and mortality and all-cause mortality. *JAMA Intern Med.* Published online September 12, 2022. doi:10.1001/jamainternmed.2022.4000

eTable 1. Baseline characteristics of participants who wore the accelerometer and those who did not in the UK Biobank

eTable 2. Stepping-based exposure variables definition

eTable 3. Assessment of CVD and cancer incidence (fatal and non-fatal) and definition of diseases

eTable 4. Tabulation of the number of events per physical activity-related cancer site considered in this study

eTable 5. Characteristics of accelerometer sample excluded from analysis

eFigure 1. A priori Defined Directed Acyclic Graph

eFigure 2. Flow diagram of participants in the study

eFigure 3. Adjusted Schoenfeld residuals (daily step counts)

eFigure 4. Dose–response association (Adjusted hazard ratios and associated 95% confidence interval band) between secondary exposures and all-cause mortality.

eFigure 5. Dose–response association (Adjusted hazard ratios and associated 95% confidence interval band) between secondary exposures and CVD mortality.

eFigure 6. Dose–response association (Adjusted hazard ratios and associated 95% confidence interval band) between secondary exposures and physical activity-related cancer composite 13 sites mortality outcome.

eFigure 7. Sequentially adjusted dose–response quintile-based absolute risk describing the associations between daily steps and mortality outcomes (events rate per 10000 person-years and associated 95% confidence intervals)

eFigure 8. Sequentially adjusted dose–response quintile-based absolute risk describing the associations between incidental steps and mortality outcomes (events rate per 10000 person-years and associated 95% confidence intervals)

eFigure 9. Sequentially adjusted dose–response quintile-based absolute risk describing the associations between purposeful steps and mortality outcomes (events rate per 10000 person-years and associated 95% confidence intervals).

eFigure 10. Sequentially adjusted dose–response quintile-based absolute risk describing the associations between peak-30 cadence and mortality outcomes (events rate per 10000 person-years and associated 95% confidence intervals)

© 2022 American Medical Association. All rights reserved.

eFigure 11. Dose–response association (Adjusted[a] hazard ratios and associated 95% confidence interval band) between primary exposures and CVD incidence.

eFigure 12. Dose-response association (Adjusteda hazard ratios and associated 95% confidence interval band) between primary exposures and physical activity-related cancer composite 13 sites incidence outcome.

eFigure 13. Dose–response association (Adjusted[a] hazard ratios and associated 95% confidence interval band) between secondary exposures and CVD incidence.

eFigure 14. Dose–response association (Adjusted[a] hazard ratios and associated 95% confidence interval band) between secondary exposures and physical activity-related cancer composite 13 sites incidence outcome.

eFigure 15. Age modification effects

eFigure 16. Dose–response association (Adjusted[a] hazard ratios and associated 95% confidence interval band) between primary exposures and all-cause mortality (Sensitivity analysis: left-truncation at 2 years follow-up).

eFigure 17. Dose–response association (Adjusted[a] hazard ratios and associated 95% confidence interval band) between primary exposures and CVD mortality (Sensitivity analysis: left-truncation at 2 years follow-up).

eFigure 18. Dose–response association (Adjusted[a] hazard ratios and associated 95% confidence interval band) between primary exposures and physical activity-related cancer composite 13 sites mortality outcome (Sensitivity analysis: left-truncation at 2 years follow-up).

eFigure 19. Dose–response association (Adjusted[a] hazard ratios and associated 95% confidence interval band) between primary exposures and CVD incidence (Sensitivity analysis: left-truncation at 2 years follow-up).

eFigure 20. Dose–response association (Adjusted[a] hazard ratios and associated 95% confidence interval band) between primary exposures and physical activity-related cancer composite 13 sites incidence outcome (Sensitivity analysis: left-truncation at 2 years follow-up).

eFigure 21. Dose–response association (Adjusted[a] hazard ratios and associated 95% confidence interval band) between primary exposures and all-cause mortality (Sensitivity analysis: addition of body mass index, and sleep and dietary score).

eFigure 22. Dose–response association (Adjusted[a] hazard ratios and associated 95% confidence interval band) between primary exposures and CVD mortality (Sensitivity analysis: addition of body mass index, and sleep and dietary score).

eFigure 23. Dose–response association (Adjusted[a] hazard ratios and associated 95% confidence interval band) between primary exposures and physical activity-related

© 2022 American Medical Association. All rights reserved.

cancer composite 13 sites mortality outcome (Sensitivity analysis: addition of body mass index, and sleep and dietary score).

eFigure 24. Dose–response association (Adjusted[a] hazard ratios and associated 95% confidence interval band) between primary exposures and CVD incidence (Sensitivity analysis: addition of body mass index, and sleep and dietary score).

eFigure 25. Dose–response association (Adjusted[a] hazard ratios and associated 95% confidence interval band) between primary exposures and physical activity-related cancer composite 13 sites incidence outcome (Sensitivity analysis: addition of body mass index, and sleep and dietary score).

eFigure 26. Dose–response association (Adjusted[a] hazard ratios and associated 95% confidence interval band) between primary exposures and all-cause mortality (Sensitivity analysis: restricted to participants with valid wear time days of 20h or more).

eFigure 27. Dose–response association (Adjusted[a] hazard ratios and associated 95% confidence interval band) between primary exposures and CVD mortality (Sensitivity analysis: restricted to participants with valid wear time days of 20h or more).

eFigure 28. Dose–response association (Adjusted[a] hazard ratios and associated 95% confidence interval band) between primary exposures and physical activity-related cancer composite 13 sites mortality outcome (Sensitivity analysis: restricted to participants with valid wear time days of 20h or more).

eFigure 29. Dose–response association (Adjusted[a] hazard ratios and associated 95% confidence interval band) between primary exposures and CVD incidence (Sensitivity analysis: restricted to participants with valid wear time days of 20h or more).

eFigure 30. Dose–response association (Adjusted[a] hazard ratios and associated 95% confidence interval band) between primary exposures and physical activity-related cancer composite 13 sites incidence outcome (Sensitivity analysis: restricted to participants with valid wear time days of 20h or more).

This supplemental material has been provided by the authors to give readers additional information about their work.

© 2022 American Medical Association. All rights reserved.

eTable 1. Baseline characteristics of participants who wore the accelerometer and those who did not in the UK Biobank		
	Accelerometer wearing	
	No	Yes
Sample size	398808	103684
Age (years)	56.65 (8.16)	56.05 (7.84)
Sex = male (%)	183714 (46.1)	45401 (43.8)
Ethnicity = non-white (%)	49265 (12.4)	9755 (9.4)
Education = university/college (%)	272178 (68.2)	58031 (56.0)
Townsend Index of Deprivation (lower scores indicate more affluence)	-1.19 (3.15)	-1.71 (2.83)
Smoking (%)		
Never	214554 (54.2)	58962 (57.0)
Previous	135883 (34.3)	37168 (35.9)
Current	45700 (11.5)	7277 (7.0)
Alcohol use (%)[a]		
Never	19381 (4.9)	3004 (2.9)
Previous	15244 (3.9)	2858 (2.8)
Occasional	92832 (23.6)	21029 (20.4)
Within guidelines	126547 (32.1)	37598 (36.5)
Double guidelines	81874 (20.8)	24180 (23.4)
Above double guidelines	58008 (14.7)	14465 (14.0)
Fruit consumption (servings/day)	3.05 (2.64)	3.21 (2.50)
Vegetable consumption (servings/day)	4.92 (3.41)	4.91 (3.17)
Family history of CVD = yes (%)	212025 (53.2)	56790 (54.8)
Family history of Cancer = yes (%)	100692 (25.2)	26028 (25.1)
Cholesterol medication = yes (%)	75238 (18.9)	14900 (14.4)
Insulin medication = yes (%)	5534 (1.4)	716 (0.7)
Hypertension medication = yes (%)	40151 (10.1)	7766 (7.5)
Body mass index (Kg/m^2)	27.62 (4.85)	26.73 (4.55)
Self-reported health (0-4)	2.16 (0.79)	1.99 (0.71)
Values represent Mean (SD) unless specified otherwise.<br>CVD, Cardiovascular disease		

© 2022 American Medical Association. All rights reserved.

[a]Guidelines for alcohol use in the UK recommend no more than 14 units of alcohol per week for both men and women.

© 2022 American Medical Association. All rights reserved.

eTable 2. Stepping-based exposure variables definition

Variable	Definition
Total steps per day	Average of steps accumulated in a day.
Walking steps per day	Average of steps accumulated in a day while walking (i.e. excludes running).
Peak 30-minute cadence[1,2]	Average steps/minute recorded for the 30 highest, but not necessarily consecutive, minutes in a day.
Incidental steps	Total daily steps at 1-39 steps/minute.
Purposeful steps	Total daily steps at ≥40 steps/minute.
Light intensity	Total daily steps at <100 steps/minute.
Moderate intensity	Total daily steps at ≥100 and < 130 steps/minute.
Vigorous intensity	Total daily steps at ≥130 steps/minute.
Moderate-to-vigorous intensity	Total daily steps at ≥100 steps/minute.

1.Lee IM, Shiroma EJ, Kamada M, Bassett DR, Matthews CE, Buring JE. Association of Step Volume and Intensity With All-Cause Mortality in Older Women. JAMA Intern Med. Published online May 29, 2019. doi:10.1001/jamainternmed.2019.0899
2.Tudor-Locke C, Ducharme SW, Aguiar EJ, et al. Walking cadence (steps/min) and intensity in 41 to 60-year-old adults: the CADENCE-adults study. Int J Behav Nutr Phys Act. 2020;17(1):137.

© 2022 American Medical Association. All rights reserved.

eTable 3. Assessment of CVD and cancer incidence (fatal and non-fatal) and definition of diseases	
Variable	Definition
Inpatient hospitalisation	The inpatient hospitalization data were provided by either the Hospital Episode Statistics for England, the Patient Episode Database for Wales, or the Scottish Morbidity Record for Scotland
Cancer registry	Cancer data linkage was obtained through national cancer registries. For England and Wales, cancer diagnosis data were provided by the Medical Research Information Service, based at the National Health Service Information Centre. For Scotland, cancer diagnosis data were provided by the Information Services Division, which is part of NHS Scotland
Cardiovascular disease definition	CVD was defined as coronary heart disease, stroke, and heart failure using ICD-10 classification: I0, I11, I13, I20-I51, I60-I69.
Cancer definition	Physical activity-related cancer was defined as 13 site specific cancers (esophageal adenocarcinoma, liver, lung, kidney, gastric cardia, endometrial, myeloid leukemia, myeloma, colon, head and neck, rectal, bladder, and breast) using ICD-10 classifications: C0-C3, C5-C9, C40-C42, C45-C49.

© 2022 American Medical Association. All rights reserved.

eTable 4. Tabulation of the number of events per physical activity-related[3] cancer site considered in this study

	Fatal	Non-fatal
Esophageal adenocarcinoma	89	65
Liver	47	69
Lung	169	209
Kidney	37	95
Gastric Cardia	9	39
Endometrial	21	130
Myeloid Leukemia	27	29
Myeloma	25	85
Colon	57	416
Head and neck	14	58
Rectal	16	72
Bladder	36	65
Breast	63	756

3. Moore SC, Lee IM, Weiderpass E, et al. Association of Leisure-Time Physical Activity With Risk of 26 Types of Cancer in 1.44 Million Adults. JAMA Intern Med. 2016;176(6):816-825.

© 2022 American Medical Association. All rights reserved.

eFigure 1. A priori Defined Directed Acyclic Graph

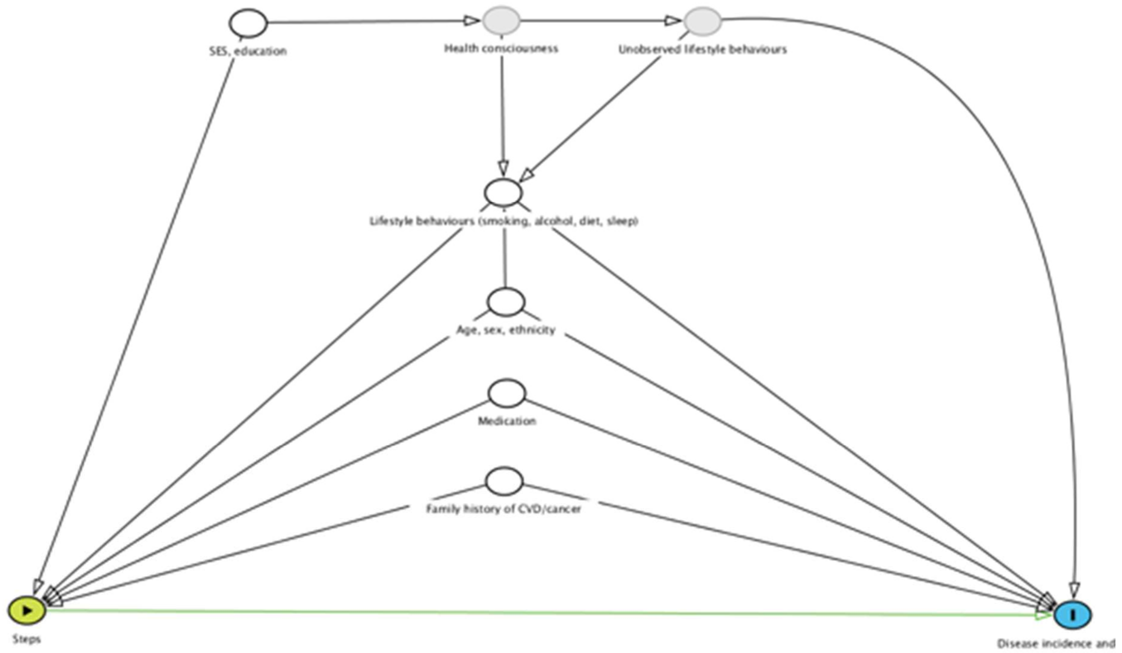

SES, socioeconomical status. The directed acyclic graph above was generated via an open-source application (Johannes Textor, Benito van der Zander, Mark K. Gilthorpe, Maciej Liskiewicz, George T.H. Ellison. Robust causal inference using directed acyclic graphs: the R package 'dagitty'. *International Journal of Epidemiology* 45(6):1887-1894, 2016. https://doi.org/10.1093/ije/dyw341).

© 2022 American Medical Association. All rights reserved.

eFigure 2. Flow diagram of participants in the study

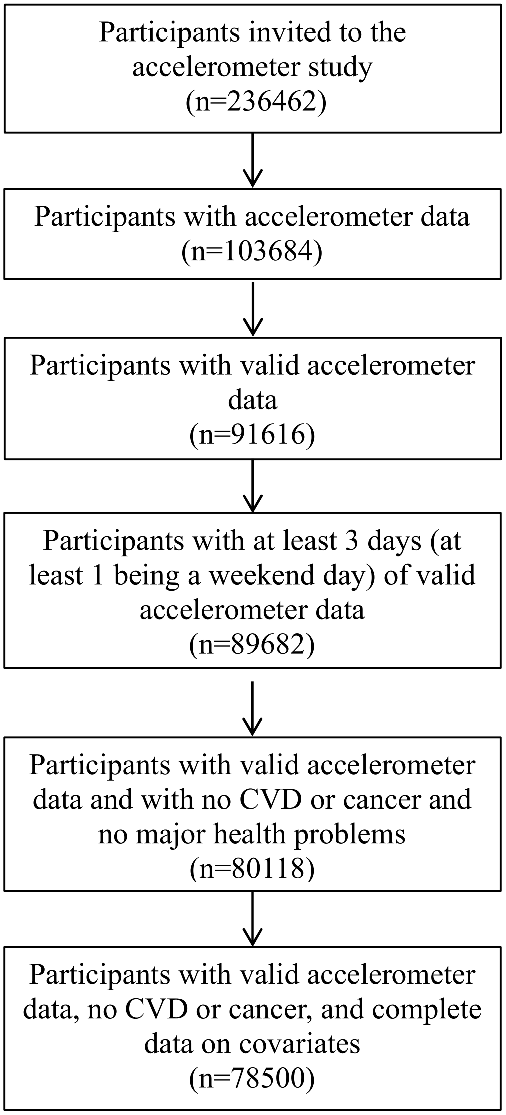

© 2022 American Medical Association. All rights reserved.

eTable 5. Characteristics of accelerometer sample excluded from analysis*	
Sample size	25184
Age (years)	61.51 (7.92)
Sex = male (%)	10264 (40.8)
Ethnicity = non-white (%)	2669 (10.6)
Education = university/college (%)	
Townsend Index of Deprivation (lower scores indicate more affluence)	-1.6 (2.9)
Smoking (%)	
Never	13794 (55.4)
Previous	9259 (37.2)
Current	1854 (7.4)
Alcohol use (%)[a]	
Never	808 (3.3)
Previous	766 (3.1)
Occasional	5169 (21.0)
Within guidelines	8854 (35.9)
Double guidelines	5661 (23.0)
Above double guidelines	3376 (13.7)
Fruit consumption (servings/day)	3.2 (2.6)
Vegetable consumption (servings/day)	5.0 (3.3)
Family history of CVD = yes (%)	13,777 (54.7)
Family history of Cancer = yes (%)	6431 (25.5)
Cholesterol medication = yes (%)	3968 (15.8)
Insulin medication = yes (%)	187 (0.7)
Hypertension medication = yes (%)	2173 (8.6)
Sleep (accelerometer-measured, min/day)	408.8 (100.4)
Accelerometer wear days	6.6 (1.0)
Total steps per day	6915.06 (4,645.52)
Walking steps per day	5817.73 (3,389.56)
Incidental steps per day	3185.80 (1,324.19)
Purposeful steps per day	2631.93 (2,328.58)
LIPA steps per day	6256.94 (3,429.85)
MPA steps per day	556.96 (742.07)
VPA steps per day	727.38 (1,598.02)
MVPA steps per day	1284.34 (2124.28)
Peak 30-min cadence (steps/min)	36.3 (15.6)

© 2022 American Medical Association. All rights reserved.

*Due to missing data, values may not equal total sample size entered in the main analyses

© 2022 American Medical Association. All rights reserved.

eFigure 3. Adjusted schoenfeld residuals (daily step counts)

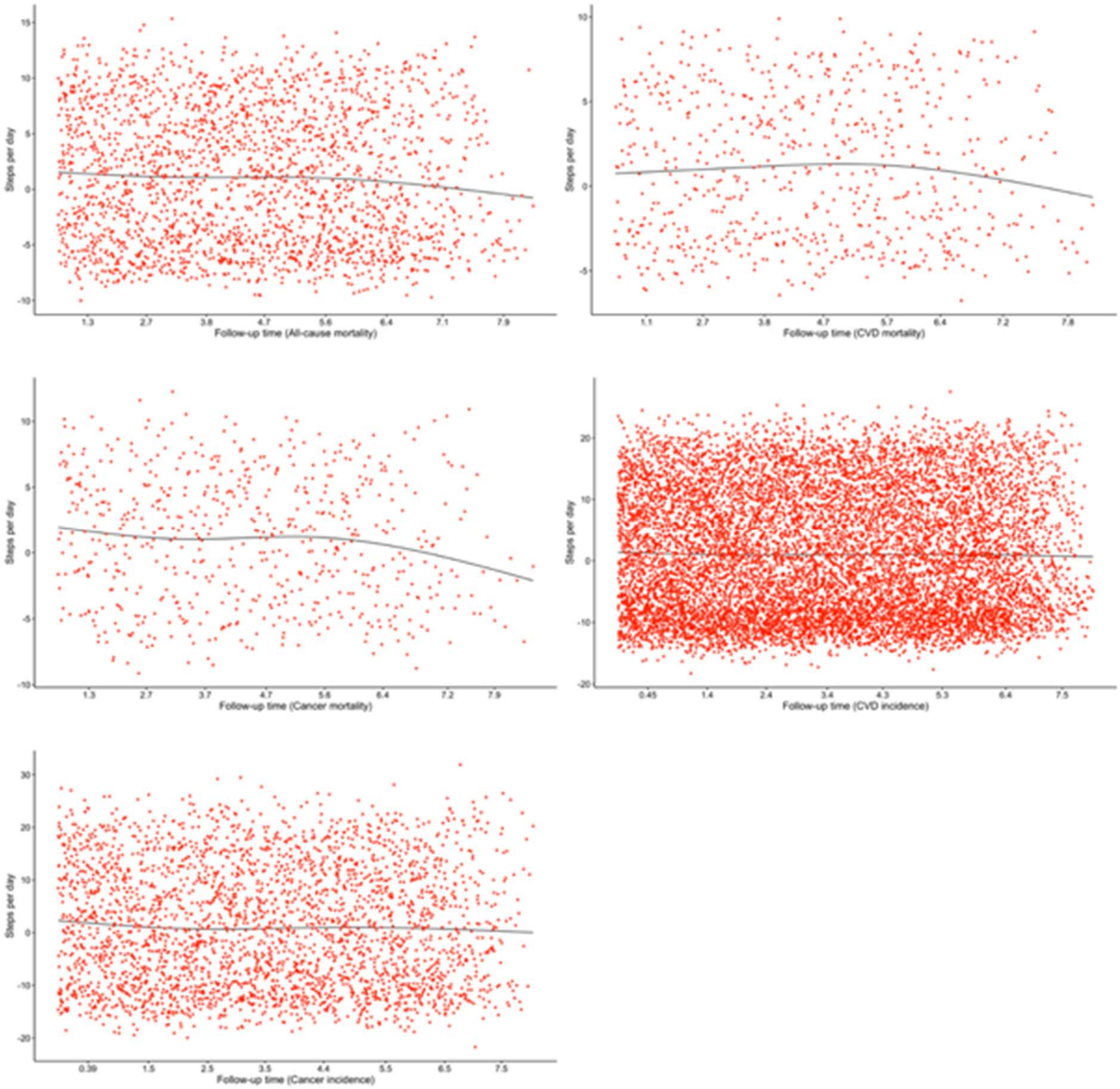

© 2022 American Medical Association. All rights reserved.

eFigure 4. Dose–response association (Adjusted[a] hazard ratios and associated 95% confidence interval band) between secondary exposures and all-cause mortality.

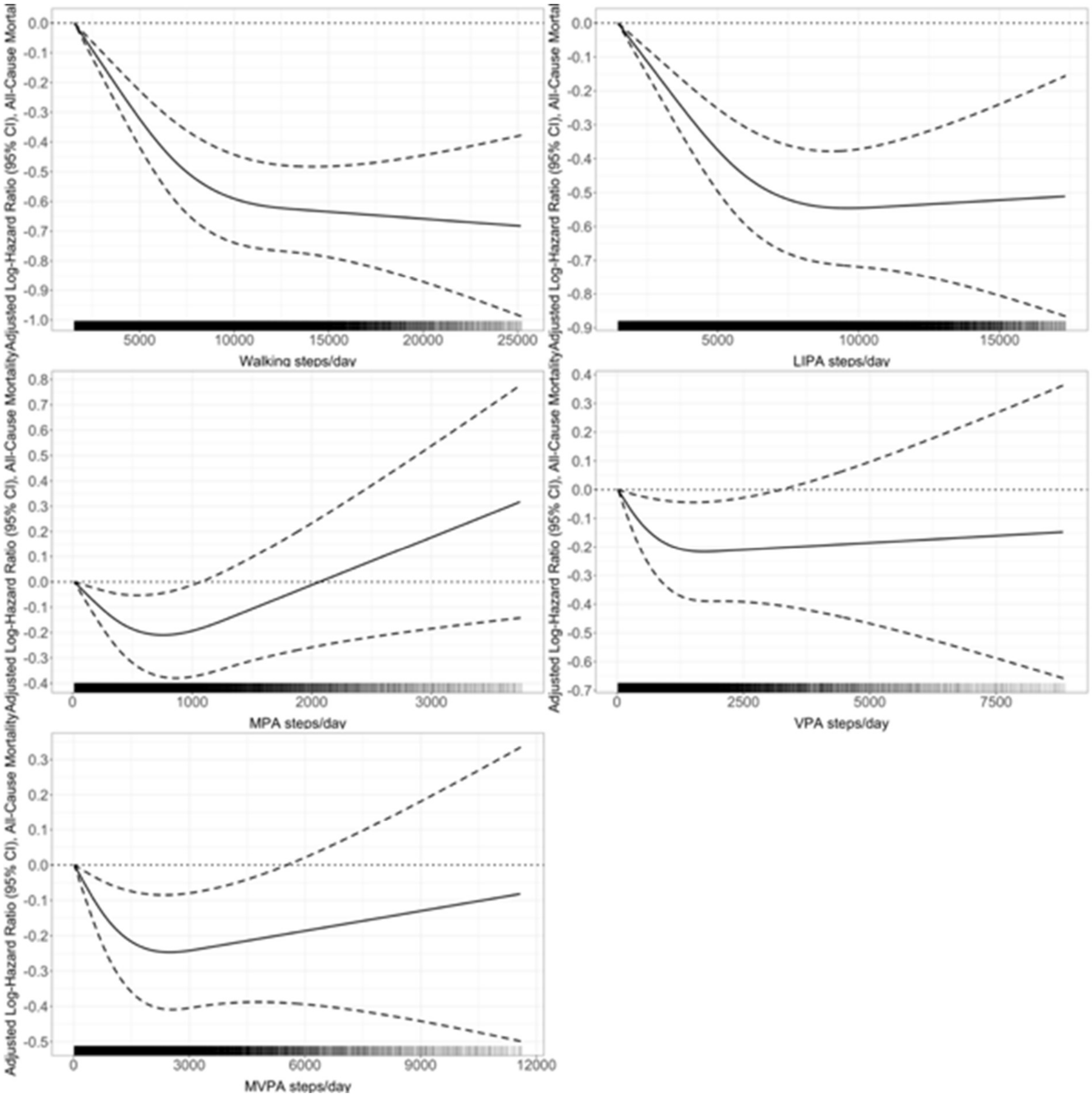

[a]Adjusted for age, sex, ethnicity, education, Townsend deprivation index, smoking, alcohol use, fruit and vegetable consumption, family history of CVD and Cancer, medication use (cholesterol, insulin, and hypertension), accelerometer-measured sleep, and wear accelerometer days. For LIPA steps, models were further adjusted for MPA and VPA steps (and vice versa). For MVPA steps, models were adjusted for LIPA steps. CVD, Cardiovascular disease; LIPA, Light intensity physical activity; MPA, Moderate intensity physical activity; VPA, Vigorous intensity physical activity; MVPA, Moderate-to-vigorous physical activity; LIPA steps/day, Total daily steps at <100 steps/minute; MPA steps/day, Total daily steps at ≥100 and < 130 steps/minute; MVPA steps/day, Total daily steps at ≥130 steps/minute; MVPA steps/day, Total daily steps at ≥100 steps/minute. Dose-response associations were assessed with restricted cubic splines with knots at 10th, 50th, and 90th centiles of the distribution of the exposure of interest. Darker colors in the lower bars represent a higher sample clustering.

© 2022 American Medical Association. All rights reserved.

eFigure 5. Dose–response association (Adjusted[a] hazard ratios and associated 95% confidence interval band) between secondary exposures and CVD mortality.

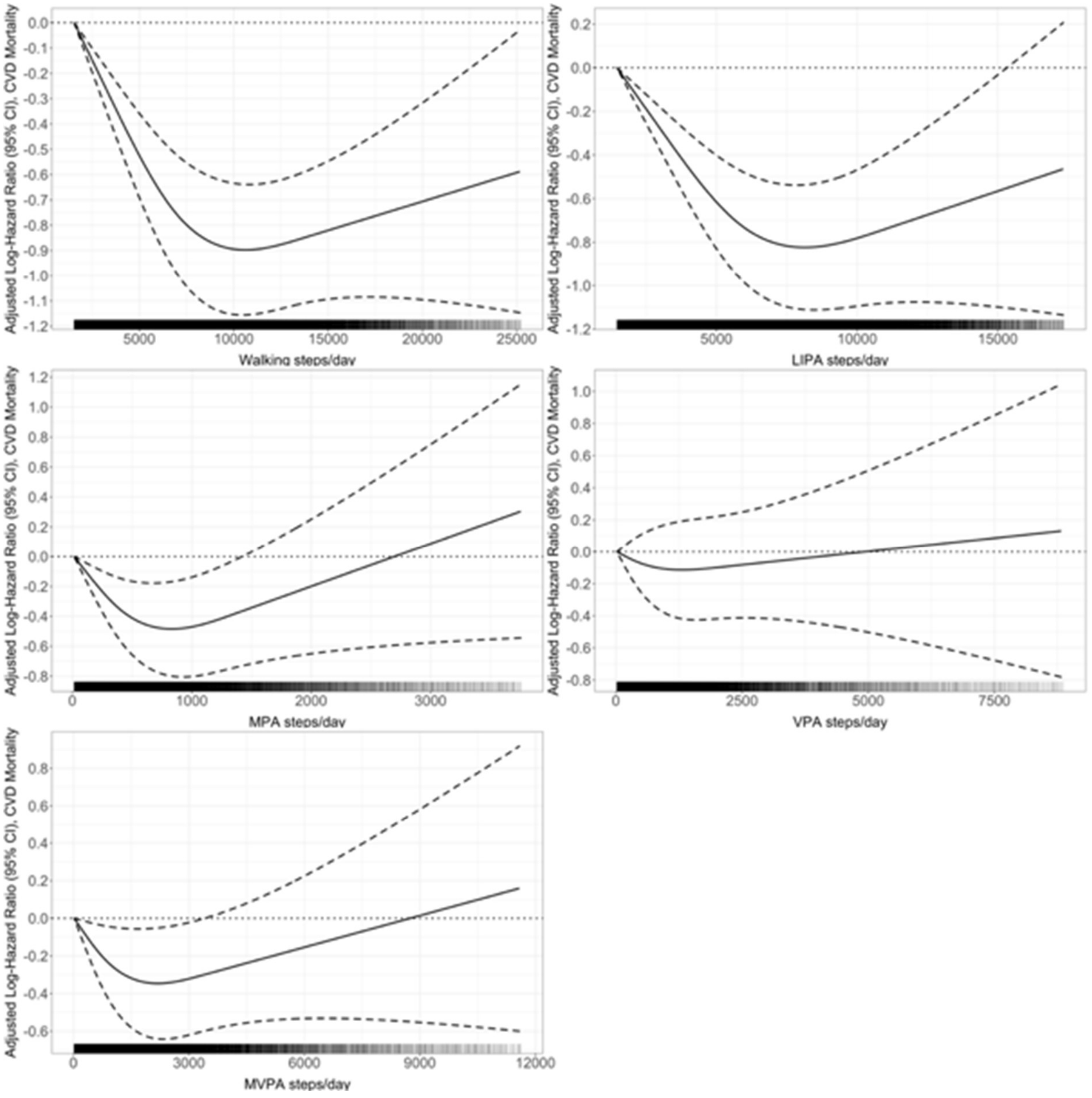

[a]Adjusted for age, sex, ethnicity, education, Townsend deprivation index, smoking, alcohol use, fruit and vegetable consumption, family history of CVD and Cancer, medication use (cholesterol, insulin, and hypertension), accelerometer-measured sleep, and wear accelerometer days. For LIPA steps, models were further adjusted for MPA and VPA steps (and vice versa). For MVPA steps, models were adjusted for LIPA steps. CVD, Cardiovascular disease; LIPA, Light intensity physical activity; MPA, Moderate intensity physical activity; VPA, Vigorous intensity physical activity; MVPA, Moderate-to-vigorous physical activity; LIPA steps/day, Total daily steps at <100 steps/minute; MPA steps/day, Total daily steps at ≥100 and < 130 steps/minute; MVPA steps/day, Total daily steps at ≥130 steps/minute; MVPA steps/day, Total daily steps at ≥100 steps/minute. Dose-response associations were assessed with restricted cubic splines with knots at 10th, 50th, and 90th centiles of the distribution of the exposure of interest. Darker colors in the lower bars represent a higher sample clustering.

© 2022 American Medical Association. All rights reserved.

eFigure 6. Dose–response association (Adjusted[a] hazard ratios and associated 95% confidence interval band) between secondary exposures and physical activity-related cancer composite 13 sites mortality outcome.

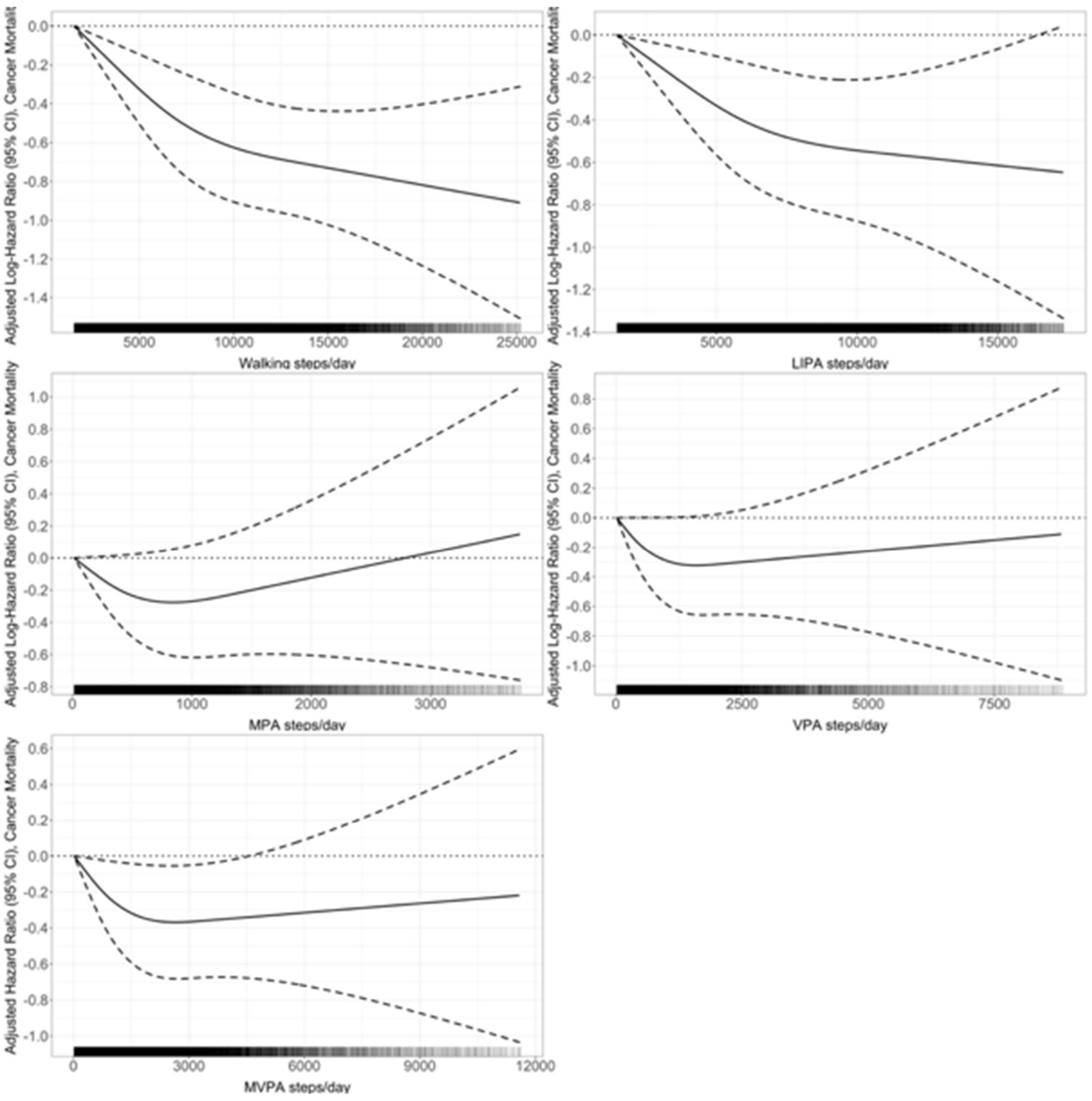

[a]Adjusted for age, sex, ethnicity, education, Townsend deprivation index, smoking, alcohol use, fruit and vegetable consumption, family history of CVD and Cancer, medication use (cholesterol, insulin, and hypertension), accelerometer-measured sleep, and wear accelerometer days. For LIPA steps, models were further adjusted for MPA and VPA steps (and vice versa). For MVPA steps, models were adjusted for LIPA steps. CVD, Cardiovascular disease; LIPA, Light intensity physical activity; MPA, Moderate intensity physical activity; VPA, Vigorous intensity physical activity; MVPA, Moderate-to-vigorous physical activity; LIPA steps/day, Total daily steps at <100 steps/minute; MPA steps/day, Total daily steps at ≥100 and < 130 steps/minute; MVPA steps/day, Total daily steps at ≥130 steps/minute; MVPA steps/day, Total daily steps at ≥100 steps/minute. Dose-response associations were assessed with restricted cubic splines with knots at 10th, 50th, and 90th centiles of the distribution of the exposure of interest. Darker colors in the lower bars represent a higher sample clustering.

© 2022 American Medical Association. All rights reserved.

eFigure 7. Sequentially adjusted dose–response quintile-based absolute risk describing the associations between daily steps and mortality outcomes (events rate per 10000 person-years and associated 95% confidence intervals).

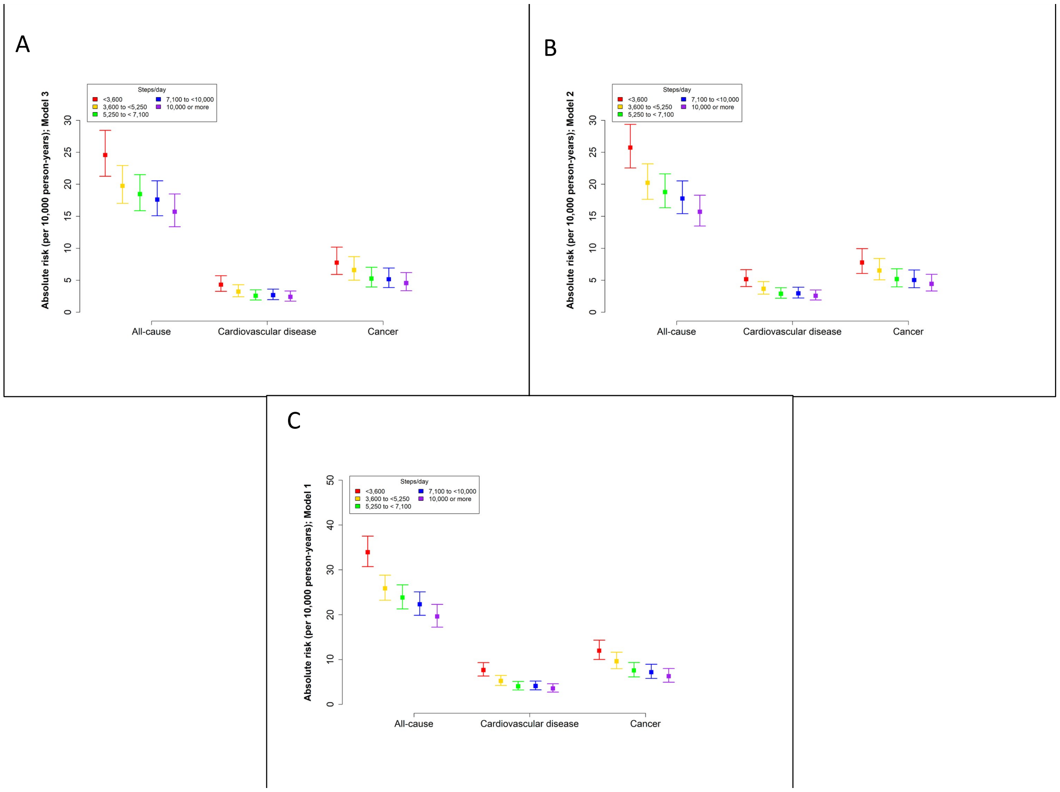

A. Model 1 adjusted for age and sex
B. Model 2 adjusted for model 1 + ethnicity, education, ses, smoking, alcohol, fruit and vegetable intake, and sleep.
C. Model 3 adjusted for model 2 + family history of CVD and cancer and medication use (cholesterol, insulin, and hypertension)

© 2022 American Medical Association. All rights reserved.

eFigure 8. Sequentially adjusted dose–response quintile-based absolute risk describing the associations between incidental steps and mortality outcomes (events rate per 10000 person-years and associated 95% confidence intervals)

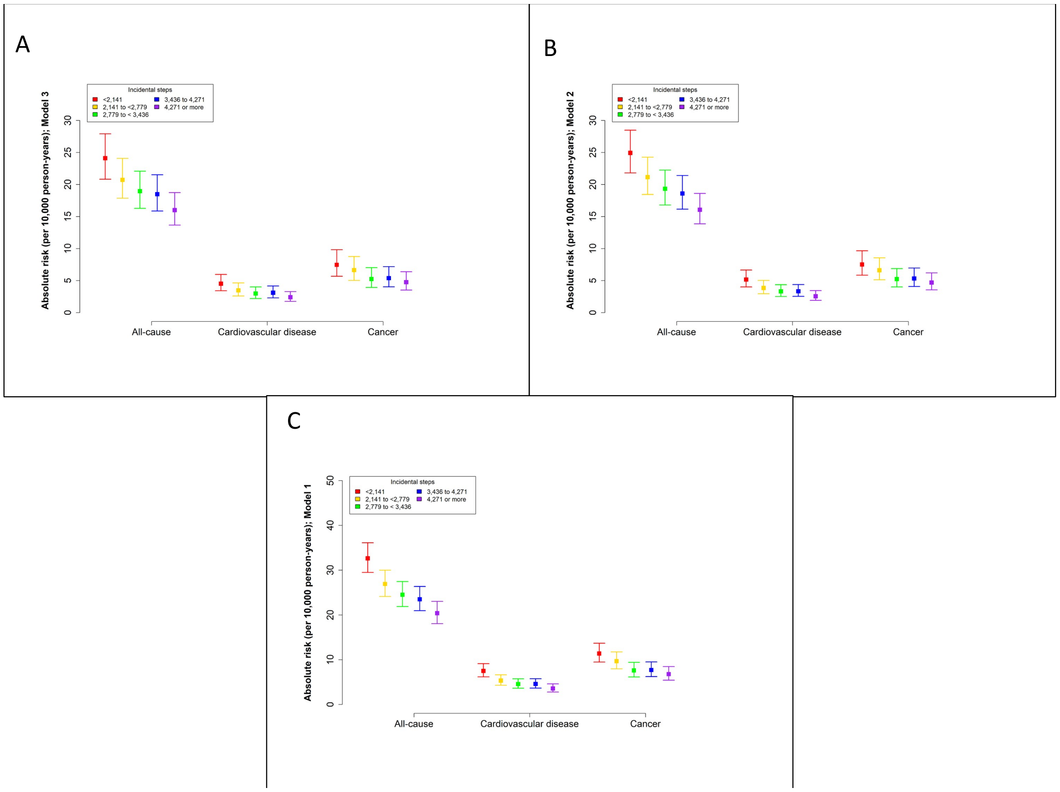

A. Model 1 adjusted for age and sex
B. Model 2 adjusted for model 1 + ethnicity, education, ses, smoking, alcohol, fruit and vegetable intake, and sleep.
C. Model 3 adjusted for model 2 + family history of CVD and cancer and medication use (cholesterol, insulin, and hypertension)

© 2022 American Medical Association. All rights reserved.

eFigure 9. Sequentially adjusted dose–response quintile-based absolute risk describing the associations between purposeful steps and mortality outcomes (events rate per 10000 person-years and associated 95% confidence intervals).

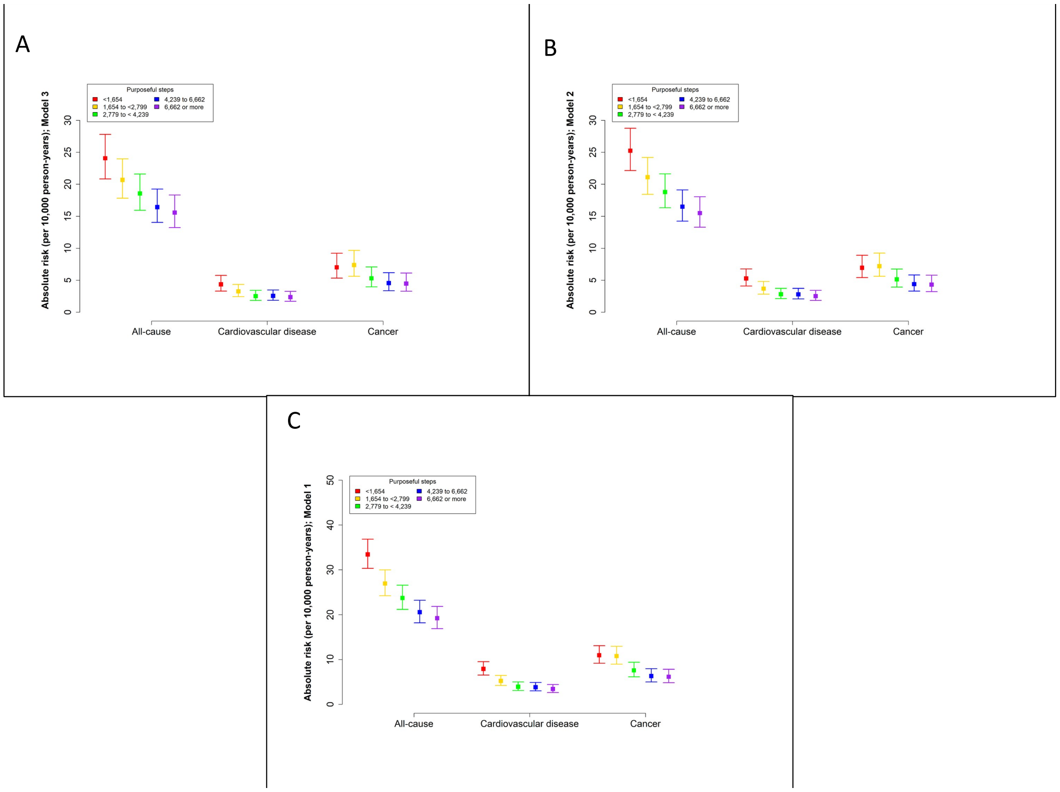

A. Model 1 adjusted for age and sex
B. Model 2 adjusted for model 1 + ethnicity, education, ses, smoking, alcohol, fruit and vegetable intake, and sleep.
C. Model 3 adjusted for model 2 + family history of CVD and cancer and medication use (cholesterol, insulin, and hypertension)

© 2022 American Medical Association. All rights reserved.

eFigure 10. Sequentially adjusted dose–response quintile-based absolute risk describing the associations between peak-30 cadence and mortality outcomes (events rate per 10000 person-years and associated 95% confidence intervals)

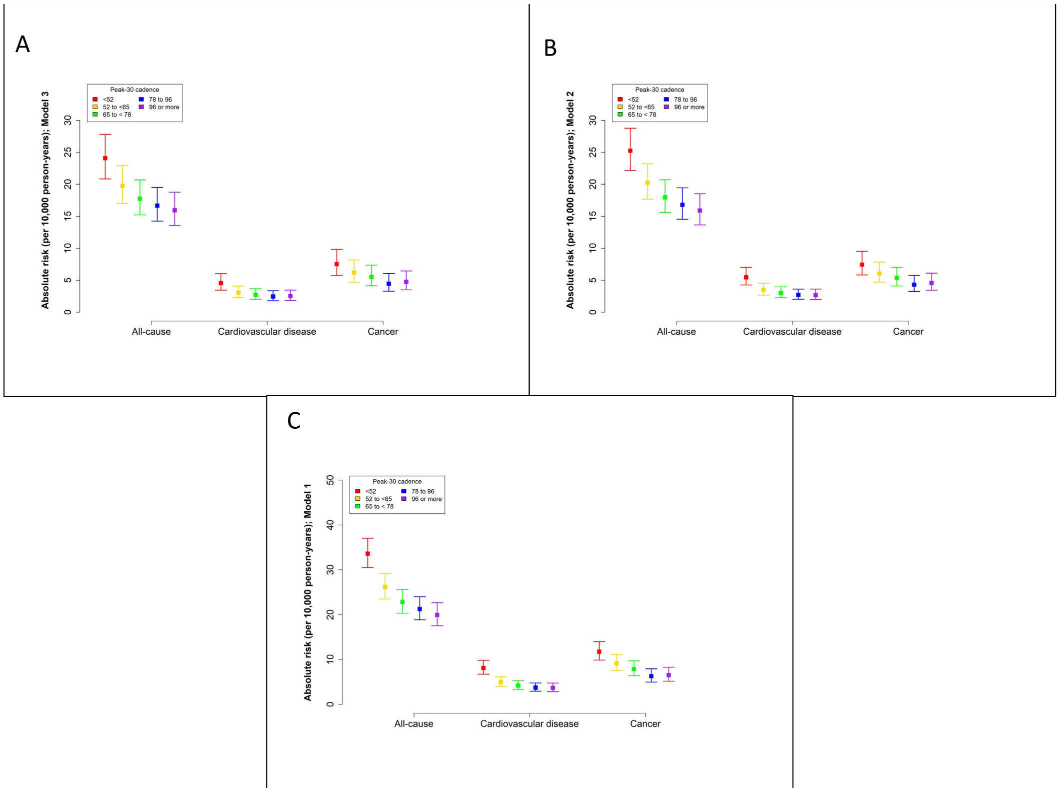

A. Model 1 adjusted for age and sex
B. Model 2 adjusted for model 1 + ethnicity, education, ses, smoking, alcohol, fruit and vegetable intake, and sleep.
C. Model 3 adjusted for model 2 + family history of CVD and cancer and medication use (cholesterol, insulin, and hypertension)

© 2022 American Medical Association. All rights reserved.

eFigure 11. Dose–response association (Adjusted[a] hazard ratios and associated 95% confidence interval band) between primary exposures and CVD incidence.

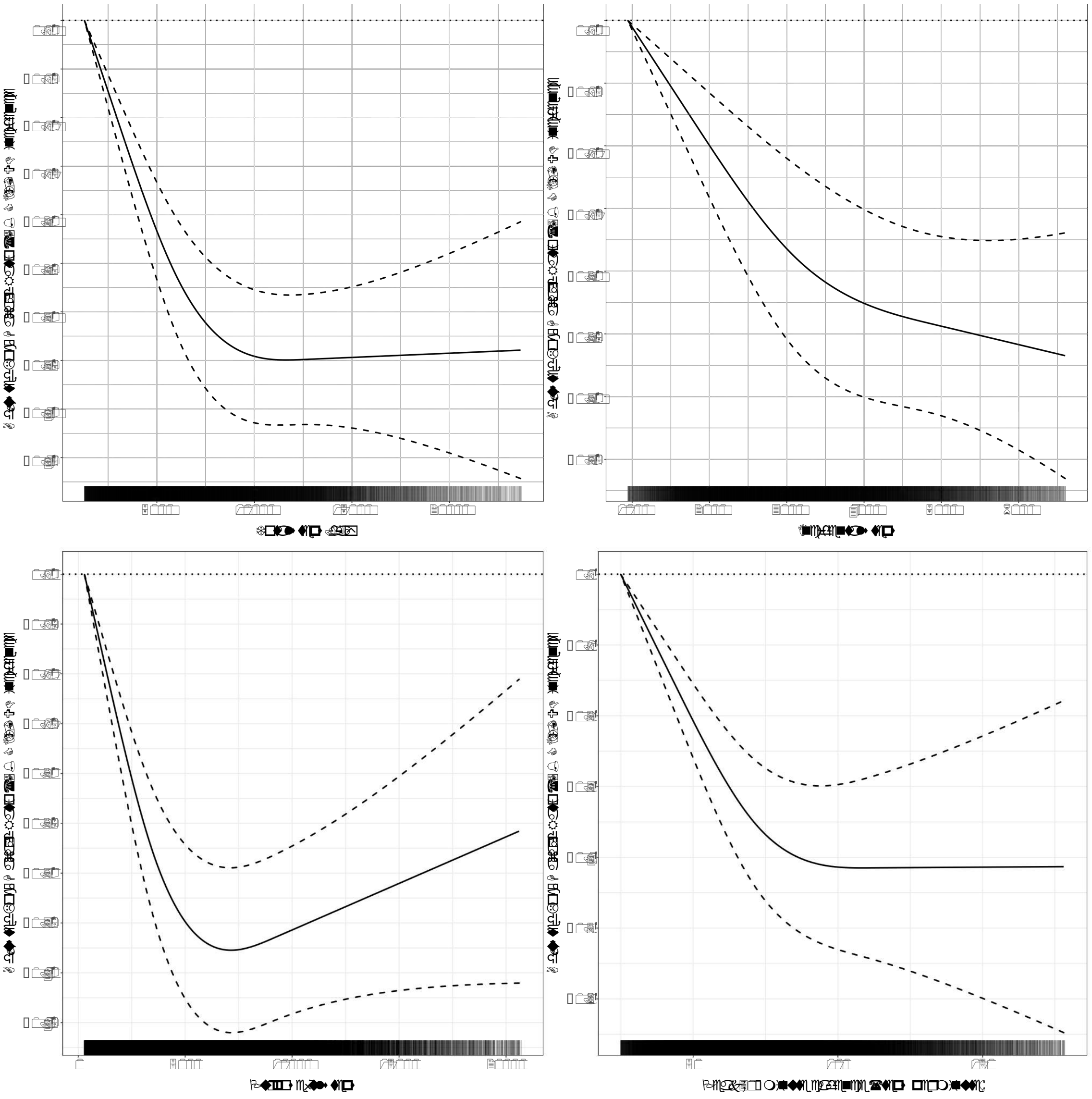

[a]Adjusted for age, sex, ethnicity, education, Townsend deprivation index, smoking, alcohol use, fruit and vegetable consumption, family history of CVD and Cancer, medication use (cholesterol, insulin, and hypertension), accelerometer-measured sleep, and wear accelerometer days. For incidental steps, models were further adjusted for purposeful steps (and vice versa). CVD, Cardiovascular disease; Total steps/day, Average of steps accumulated in a day; Incidental steps, Total daily steps at 1-39 steps/minute; Purposeful steps, Total daily steps at ≥40 steps/minute; Peak 30-min cadence, Average steps/minute recorded for the 30 highest, but not necessarily consecutive, minutes in a day. Dose-response associations were assessed with restricted cubic splines with knots at 10th, 50th, and 90th centiles of the distribution of the exposure of interest. Darker colors in the lower bars represent a higher sample clustering

© 2022 American Medical Association. All rights reserved.

eFigure 12. Dose-response association (Adjusted[a] hazard ratios and associated 95% confidence interval band) between primary exposures and physical activity-related cancer composite 13 sites incidence outcome.

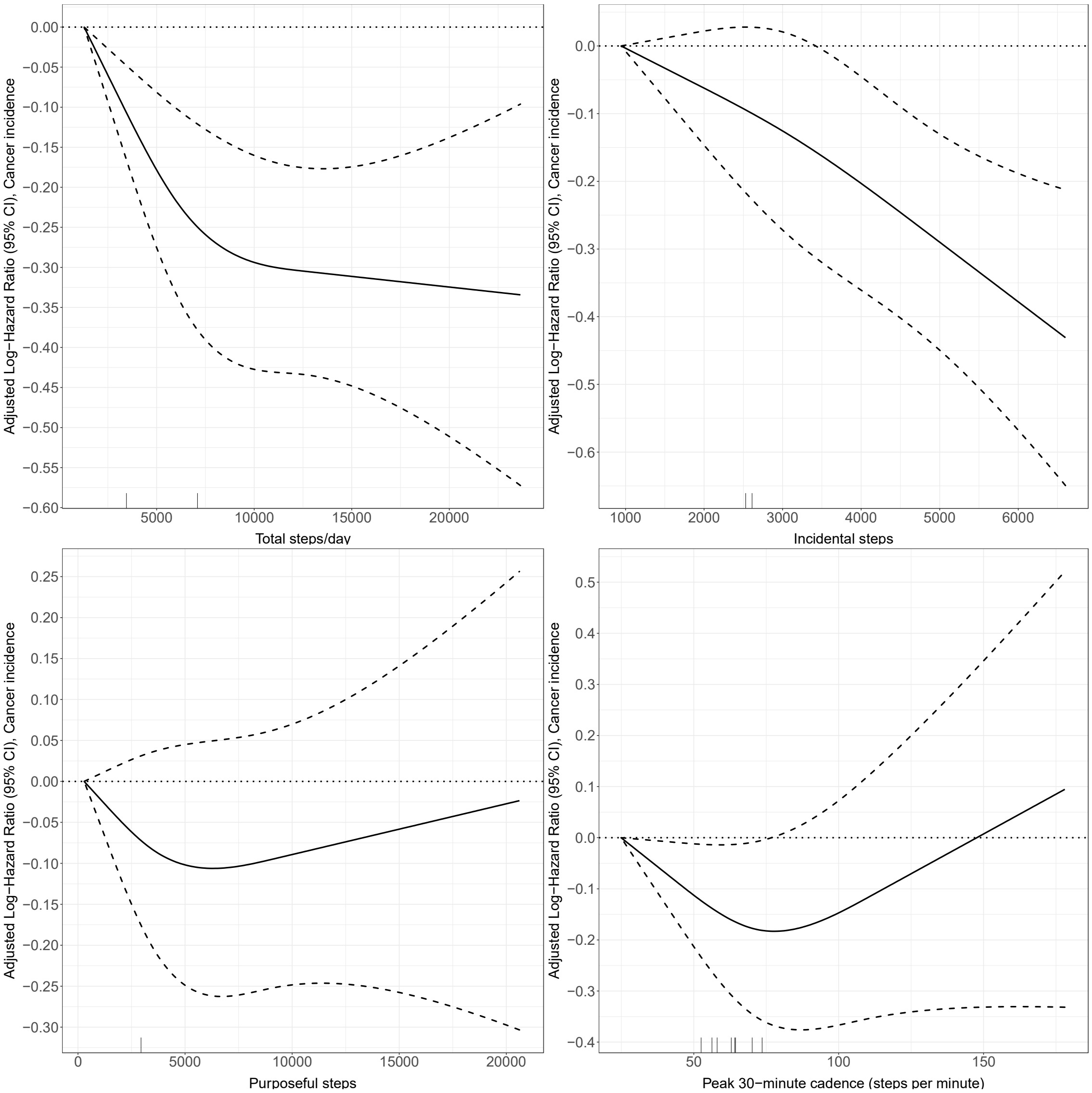

[a]Adjusted for age, sex, ethnicity, education, Townsend deprivation index, smoking, alcohol use, fruit and vegetable consumption, family history of CVD and Cancer, medication use (cholesterol, insulin, and hypertension), accelerometer-measured sleep, and wear accelerometer days. For incidental steps, models were further adjusted for purposeful steps (and vice versa). CVD, Cardiovascular disease; Total steps/day, Average of steps accumulated in a day; Incidental steps, Total daily steps at 1-39 steps/minute; Purposeful steps, Total daily steps at ≥40 steps/minute; Peak 30-min cadence, Average steps/minute recorded for the 30 highest, but not necessarily consecutive, minutes in a day. Dose-response associations were assessed with restricted cubic splines with knots at 10th, 50th, and 90th centiles of the distribution of the exposure of interest. Darker colors in the lower bars represent a higher sample clustering

© 2022 American Medical Association. All rights reserved.

eFigure 13. Dose–response association (Adjusted[a] hazard ratios and associated 95% confidence interval band) between secondary exposures and CVD incidence

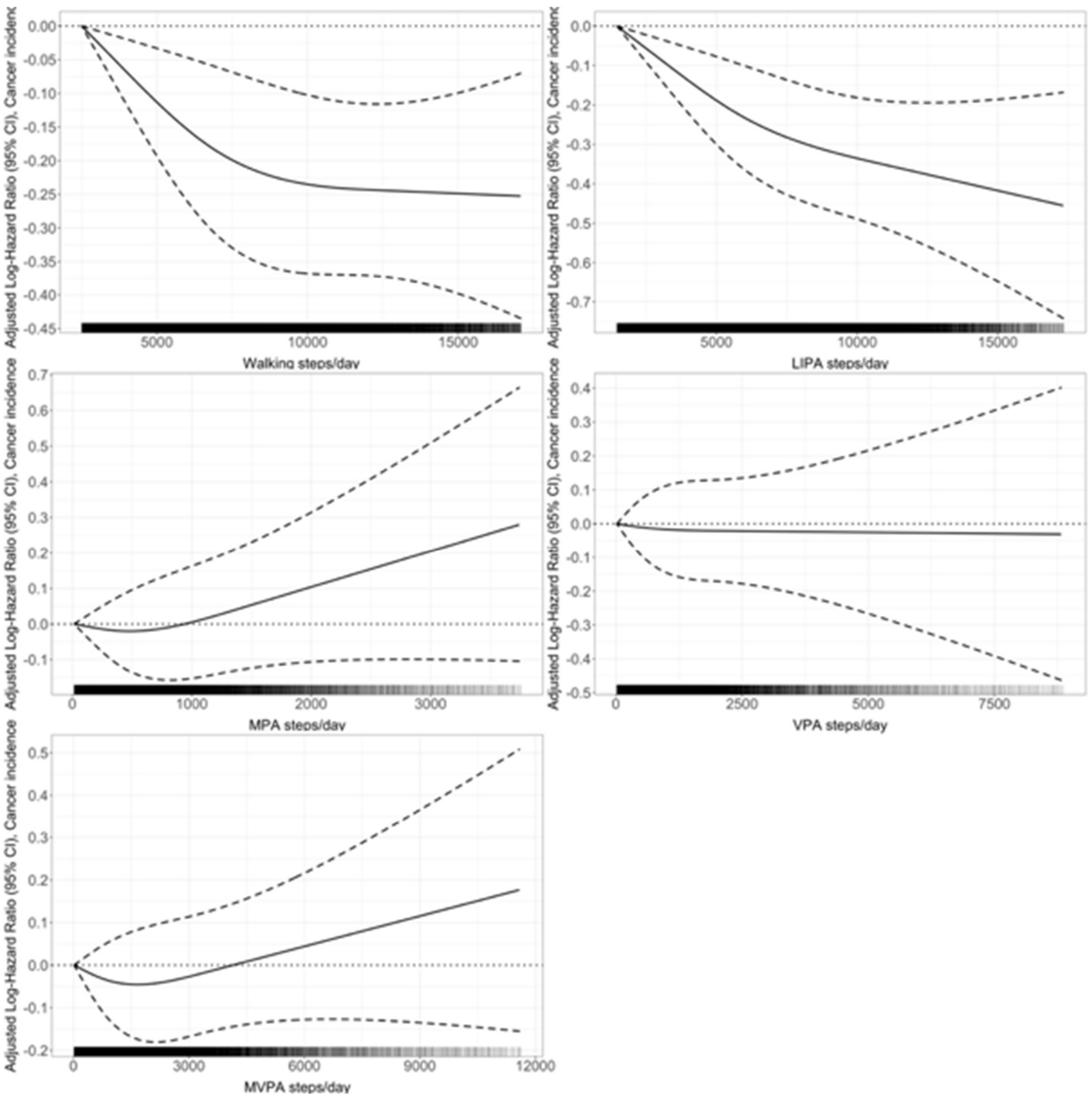

[a]Adjusted for age, sex, ethnicity, education, Townsend deprivation index, smoking, alcohol use, fruit and vegetable consumption, family history of CVD and Cancer, medication use (cholesterol, insulin, and hypertension), accelerometer-measured sleep, and wear accelerometer days. For LIPA steps, models were further adjusted for MPA and VPA steps (and vice versa). For MVPA steps, models were adjusted for LIPA steps. CVD, Cardiovascular disease; LIPA, Light intensity physical activity; MPA, Moderate intensity physical activity; VPA, Vigorous intensity physical activity; MVPA, Moderate-to-vigorous physical activity; LIPA steps/day, Total daily steps at <100 steps/minute; MPA steps/day, Total daily steps at ≥100 and < 130 steps/minute; MVPA steps/day, Total daily steps at ≥130 steps/minute; MVPA steps/day, Total daily steps at ≥100 steps/minute. Dose-response associations were assessed with restricted cubic splines with knots at 10th, 50th, and 90th centiles of the distribution of the exposure of interest. Darker colors in the lower bars represent a higher sample clustering.

© 2022 American Medical Association. All rights reserved.

eFigure 14. Dose–response association (Adjusted[a] hazard ratios and associated 95% confidence interval band) between secondary exposures and physical activity-related cancer composite 13 sites incidence outcome.

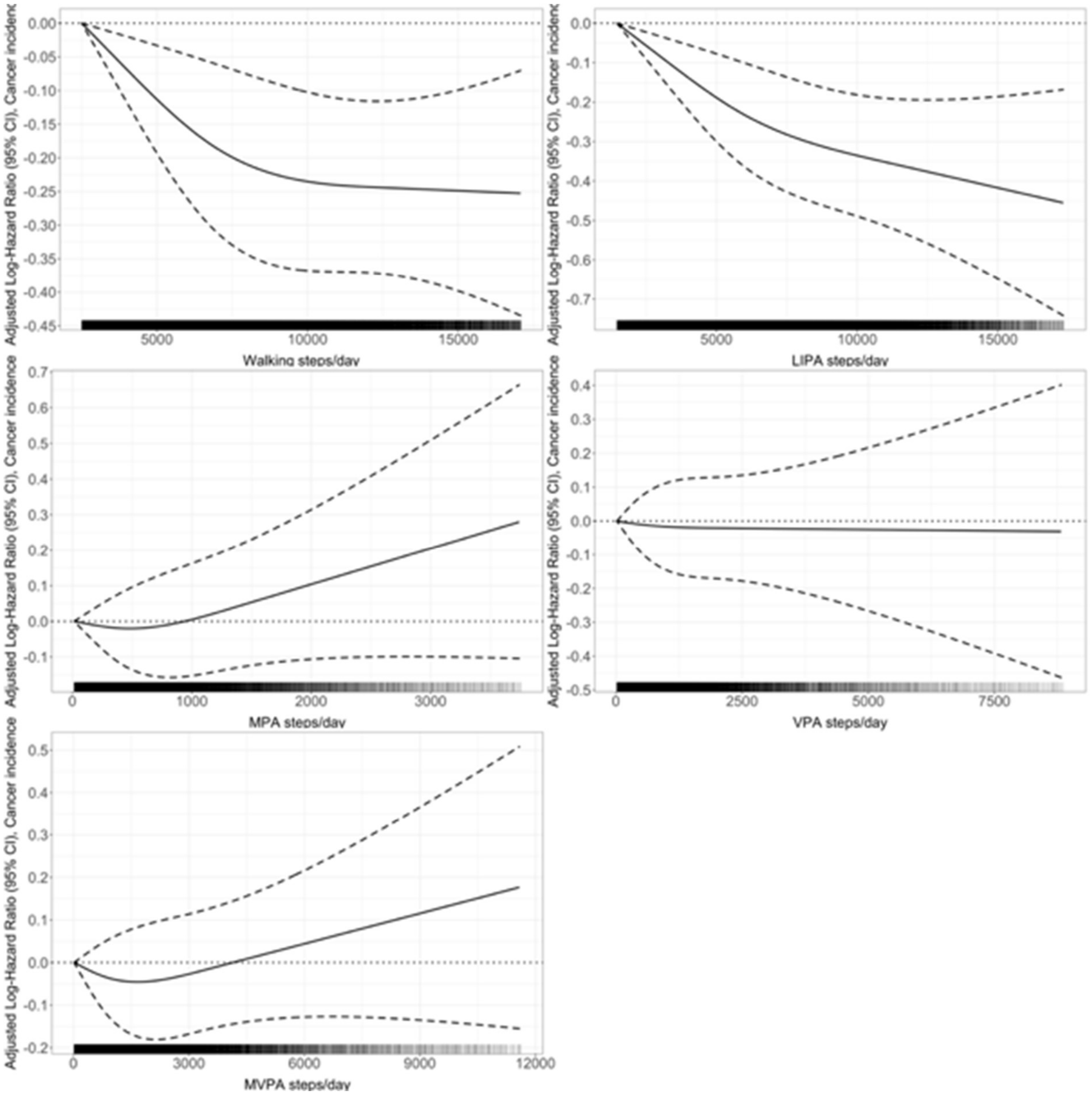

[a]Adjusted for age, sex, ethnicity, education, Townsend deprivation index, smoking, alcohol use, fruit and vegetable consumption, family history of CVD and Cancer, medication use (cholesterol, insulin, and hypertension), accelerometer-measured sleep, and wear accelerometer days. For LIPA steps, models were further adjusted for MPA and VPA steps (and vice versa). For MVPA steps, models were adjusted for LIPA steps. CVD, Cardiovascular disease; LIPA, Light intensity physical activity; MPA, Moderate intensity physical activity; VPA, Vigorous intensity physical activity; MVPA, Moderate-to-vigorous physical activity; LIPA steps/day, Total daily steps at <100 steps/minute; MPA steps/day, Total daily steps at ≥100 and < 130 steps/minute; MVPA steps/day, Total daily steps at ≥130 steps/minute; MVPA steps/day, Total daily steps at ≥100 steps/minute. Dose-response associations were assessed with restricted cubic splines with knots at 10th, 50th, and 90th centiles of the distribution of the exposure of interest. Darker colors in the lower bars represent a higher sample clustering.

© 2022 American Medical Association. All rights reserved.

eFigure 15. Age modification effects

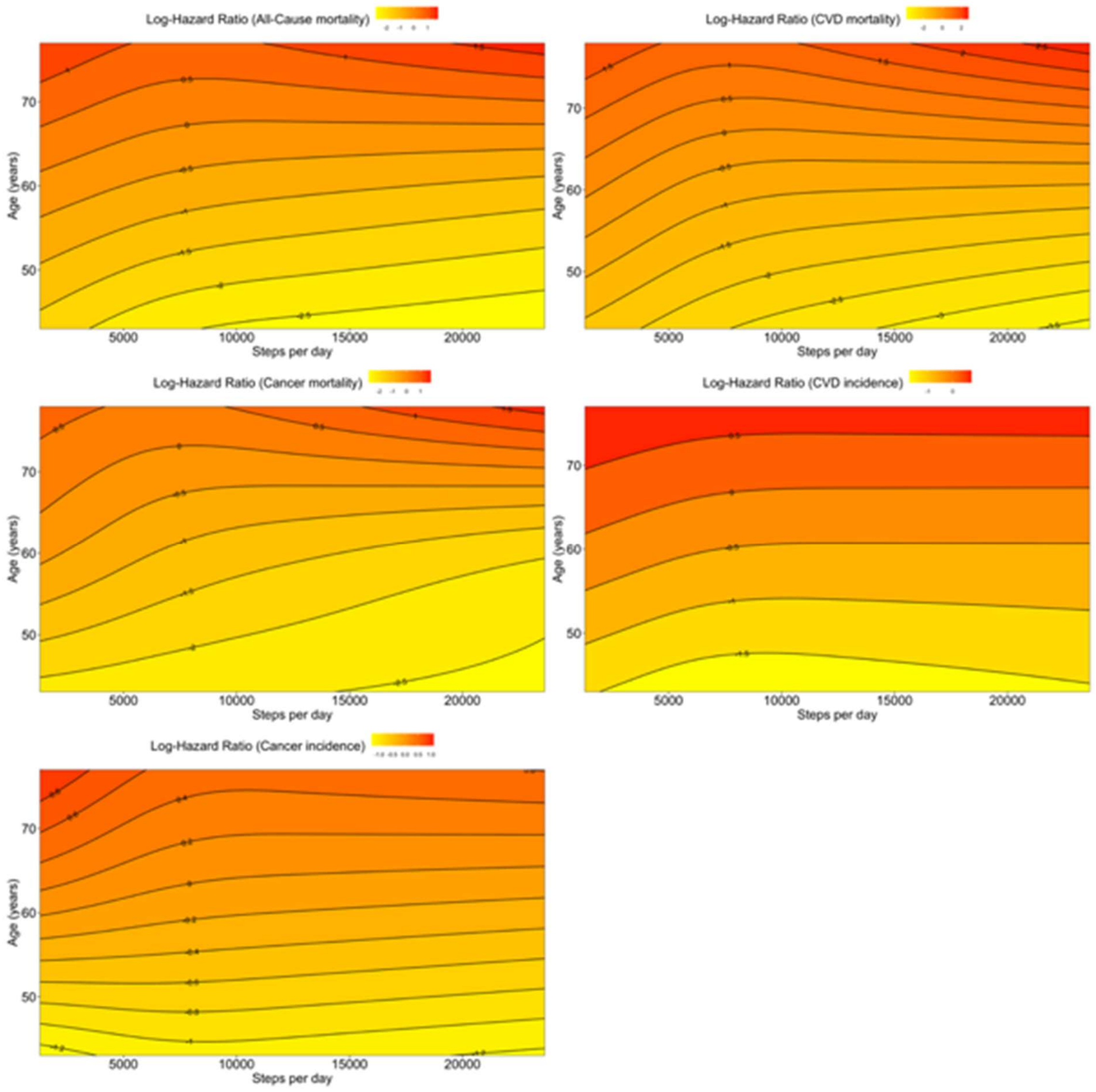

Paralel lines represent lack of interaction between age and steps per day. Darker areas represent higher risks. The lack of intereaction between age and daily steps is evident by the somewhat parallel contour lines in all plots.

© 2022 American Medical Association. All rights reserved.

eFigure 16. Dose–response association (Adjusted[a] hazard ratios and associated 95% confidence interval band) between primary exposures and all-cause mortality (Sensitivity analysis: left-truncation at 2 years follow-up).

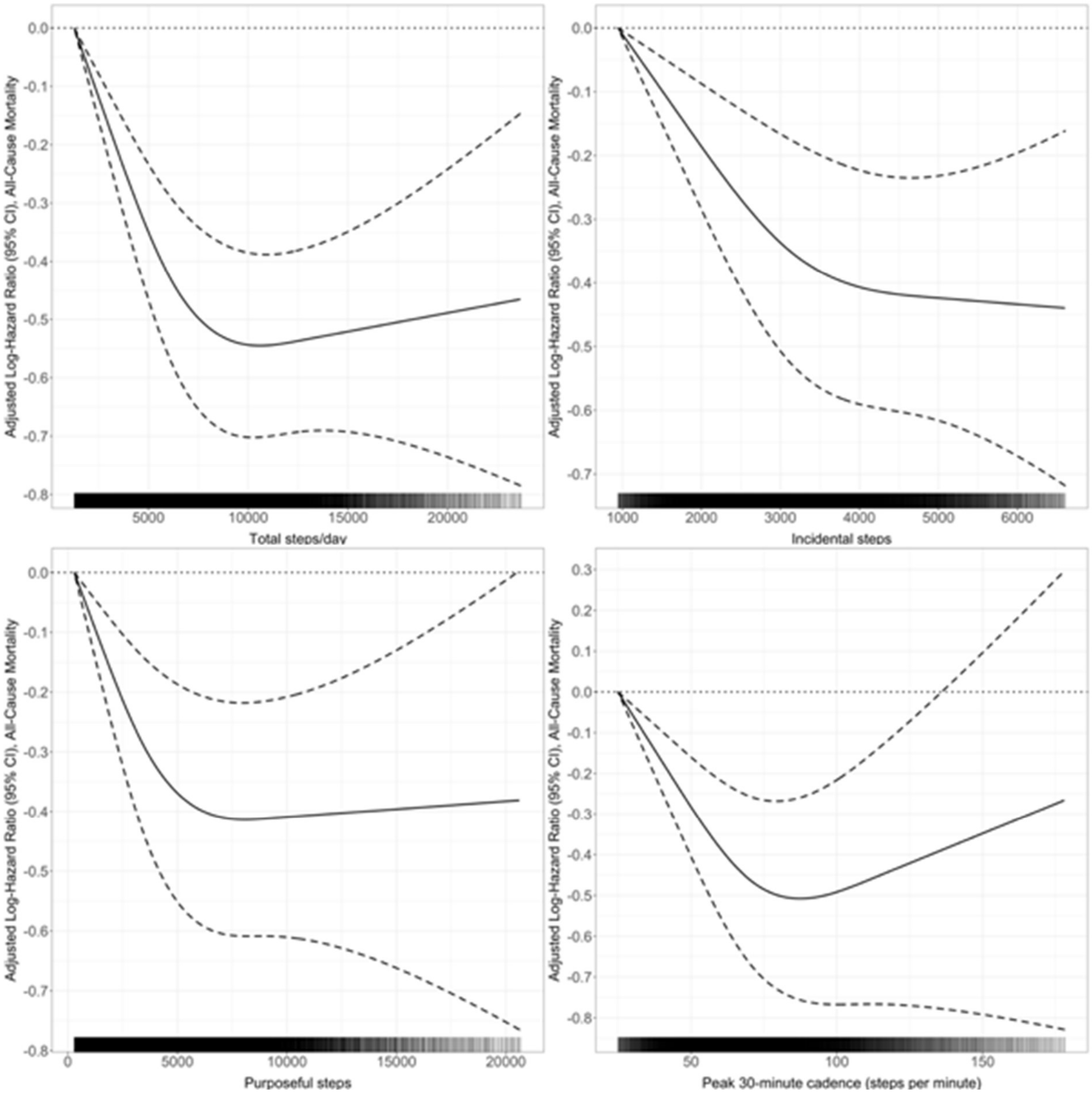

[a]Adjusted for age, sex, ethnicity, education, Townsend deprivation index, smoking, alcohol use, fruit and vegetable consumption, family history of CVD and Cancer, medication use (cholesterol, insulin, and hypertension), accelerometer-measured sleep, and wear accelerometer days. For incidental steps, models were further adjusted for purposeful steps (and vice versa). CVD, Cardiovascular disease; Total steps/day, Average of steps accumulated in a day; Incidental steps, Total daily steps at 1-39 steps/minute; Purposeful steps, Total daily steps at ≥40 steps/minute; Peak 30-min cadence, Average steps/minute recorded for the 30 highest, but not necessarily consecutive, minutes in a day. Dose-response associations were assessed with restricted cubic splines with knots at 10th, 50th, and 90th centiles of the distribution of the exposure of interest. Darker colors in the lower bars represent a higher sample clustering.

© 2022 American Medical Association. All rights reserved.

eFigure 17. Dose–response association (Adjusted[a] hazard ratios and associated 95% confidence interval band) between primary exposures and CVD mortality (Sensitivity analysis: left-truncation at 2 years follow-up).

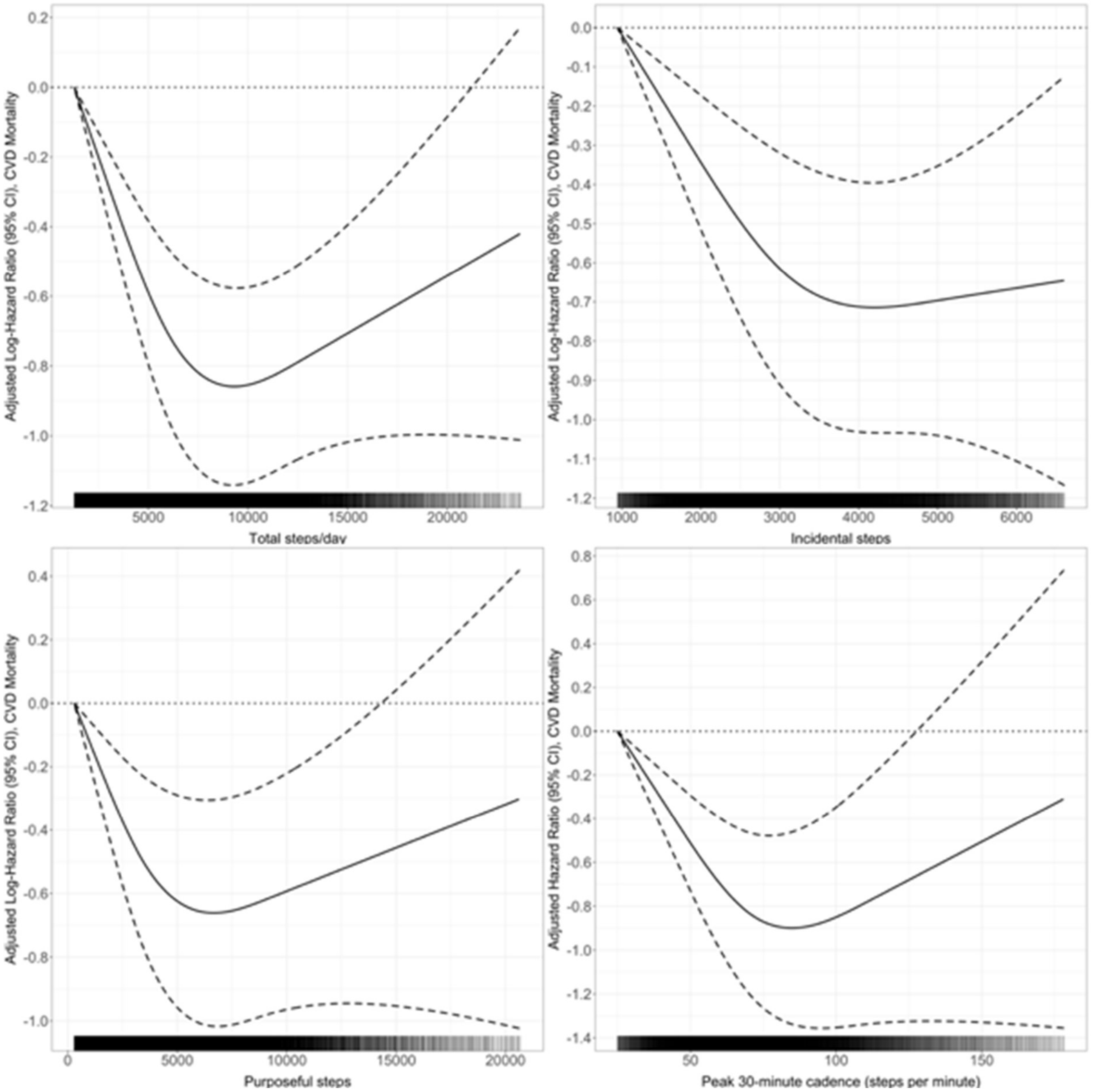

[a]Adjusted for age, sex, ethnicity, education, Townsend deprivation index, smoking, alcohol use, fruit and vegetable consumption, family history of CVD and Cancer, medication use (cholesterol, insulin, and hypertension), accelerometer-measured sleep, and wear accelerometer days. For incidental steps, models were further adjusted for purposeful steps (and vice versa). CVD, Cardiovascular disease; Total steps/day, Average of steps accumulated in a day; Incidental steps, Total daily steps at 1-39 steps/minute; Purposeful steps, Total daily steps at ≥40 steps/minute; Peak 30-min cadence, Average steps/minute recorded for the 30 highest, but not necessarily consecutive, minutes in a day. Dose-response associations were assessed with restricted cubic splines with knots at 10th, 50th, and 90th centiles of the distribution of the exposure of interest. Darker colors in the lower bars represent a higher sample clustering.

© 2022 American Medical Association. All rights reserved.

eFigure 18. Dose–response association (Adjusted[a] hazard ratios and associated 95% confidence interval band) between primary exposures and physical activity-related cancer composite 13 sites mortality outcome (Sensitivity analysis: left-truncation at 2 years follow-up).

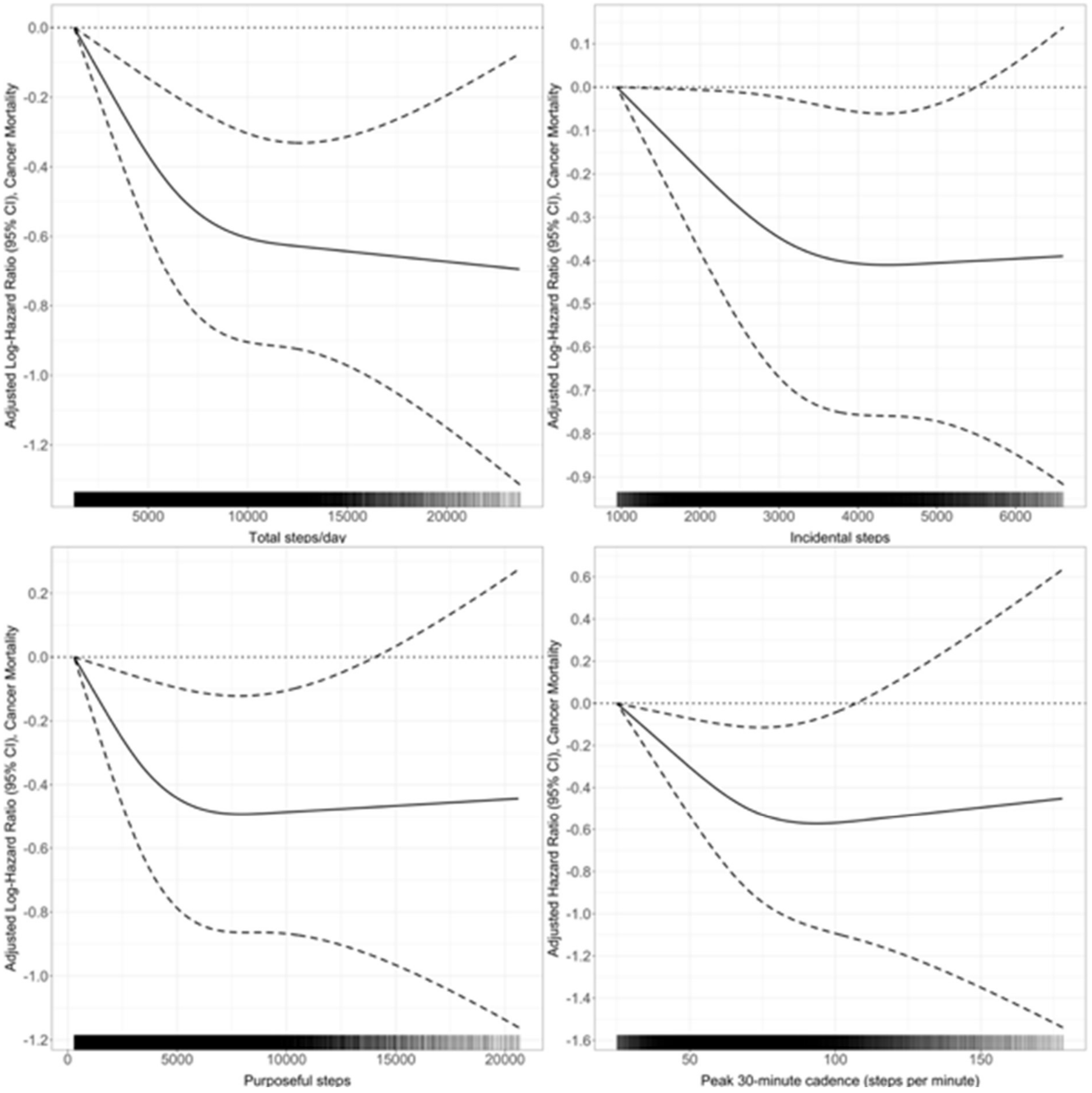

[a]Adjusted for age, sex, ethnicity, education, Townsend deprivation index, smoking, alcohol use, fruit and vegetable consumption, family history of CVD and Cancer, medication use (cholesterol, insulin, and hypertension), accelerometer-measured sleep, and wear accelerometer days. For incidental steps, models were further adjusted for purposeful steps (and vice versa). CVD, Cardiovascular disease; Total steps/day, Average of steps accumulated in a day; Incidental steps, Total daily steps at 1-39 steps/minute; Purposeful steps, Total daily steps at ≥40 steps/minute; Peak 30-min cadence, Average steps/minute recorded for the 30 highest, but not necessarily consecutive, minutes in a day. Dose-response associations were assessed with restricted cubic splines with knots at 10th, 50th, and 90th centiles of the distribution of the exposure of interest. Darker colors in the lower bars represent a higher sample clustering.

© 2022 American Medical Association. All rights reserved.

eFigure 19. Dose–response association (Adjusted[a] hazard ratios and associated 95% confidence interval band) between primary exposures and CVD incidence (Sensitivity analysis: left-truncation at 2 years follow-up).

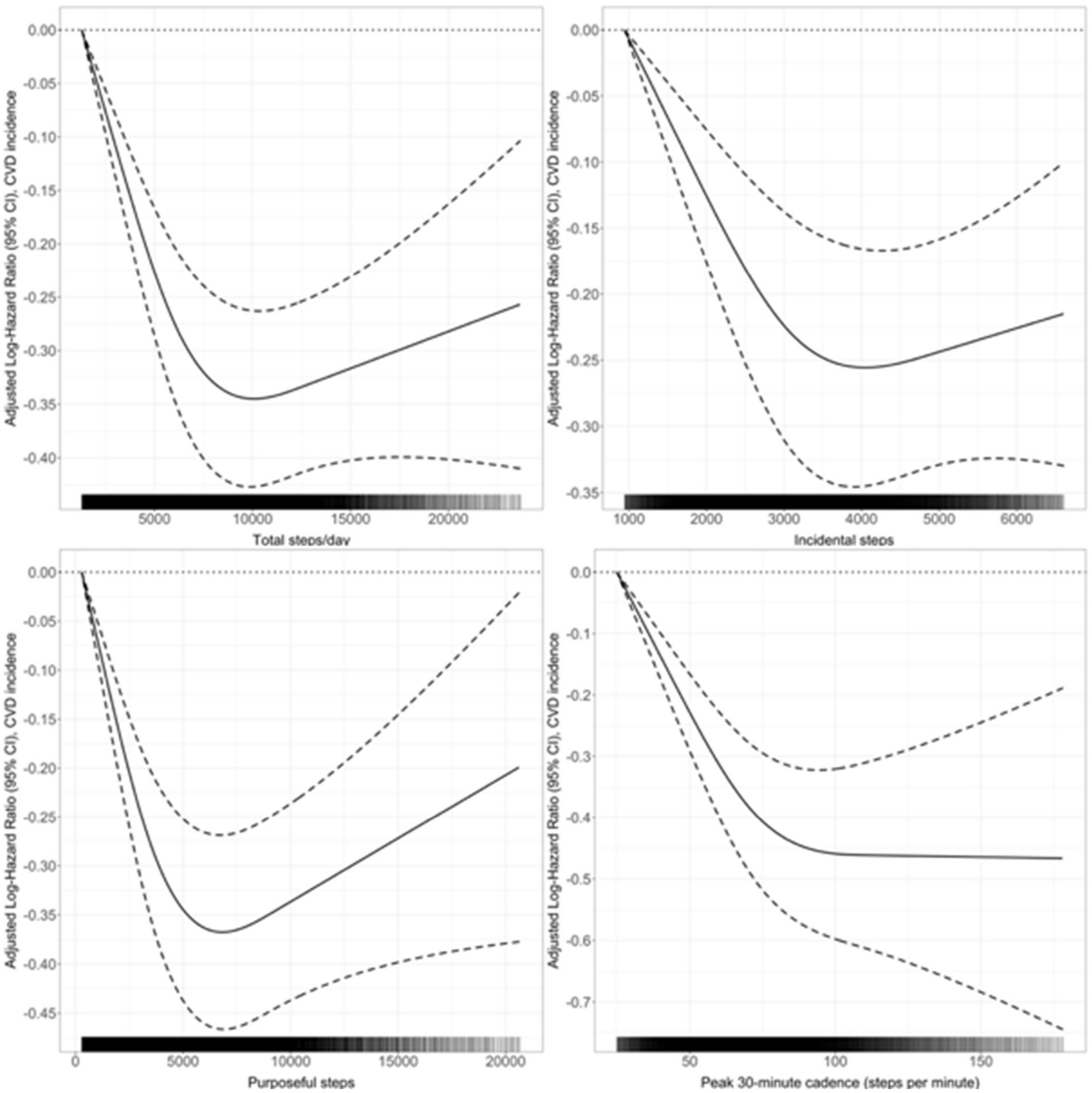

[a]Adjusted for age, sex, ethnicity, education, Townsend deprivation index, smoking, alcohol use, fruit and vegetable consumption, family history of CVD and Cancer, medication use (cholesterol, insulin, and hypertension), accelerometer-measured sleep, and wear accelerometer days. For incidental steps, models were further adjusted for purposeful steps (and vice versa). CVD, Cardiovascular disease; Total steps/day, Average of steps accumulated in a day; Incidental steps, Total daily steps at 1-39 steps/minute; Purposeful steps, Total daily steps at ≥40 steps/minute; Peak 30-min cadence, Average steps/minute recorded for the 30 highest, but not necessarily consecutive, minutes in a day. Dose-response associations were assessed with restricted cubic splines with knots at 10th, 50th, and 90th centiles of the distribution of the exposure of interest. Darker colors in the lower bars represent a higher sample clustering.

© 2022 American Medical Association. All rights reserved.

eFigure 20. Dose–response association (Adjusted[a] hazard ratios and associated 95% confidence interval band) between primary exposures and physical activity-related cancer composite 13 sites incidence outcome (Sensitivity analysis: left-truncation at 2 years follow-up).

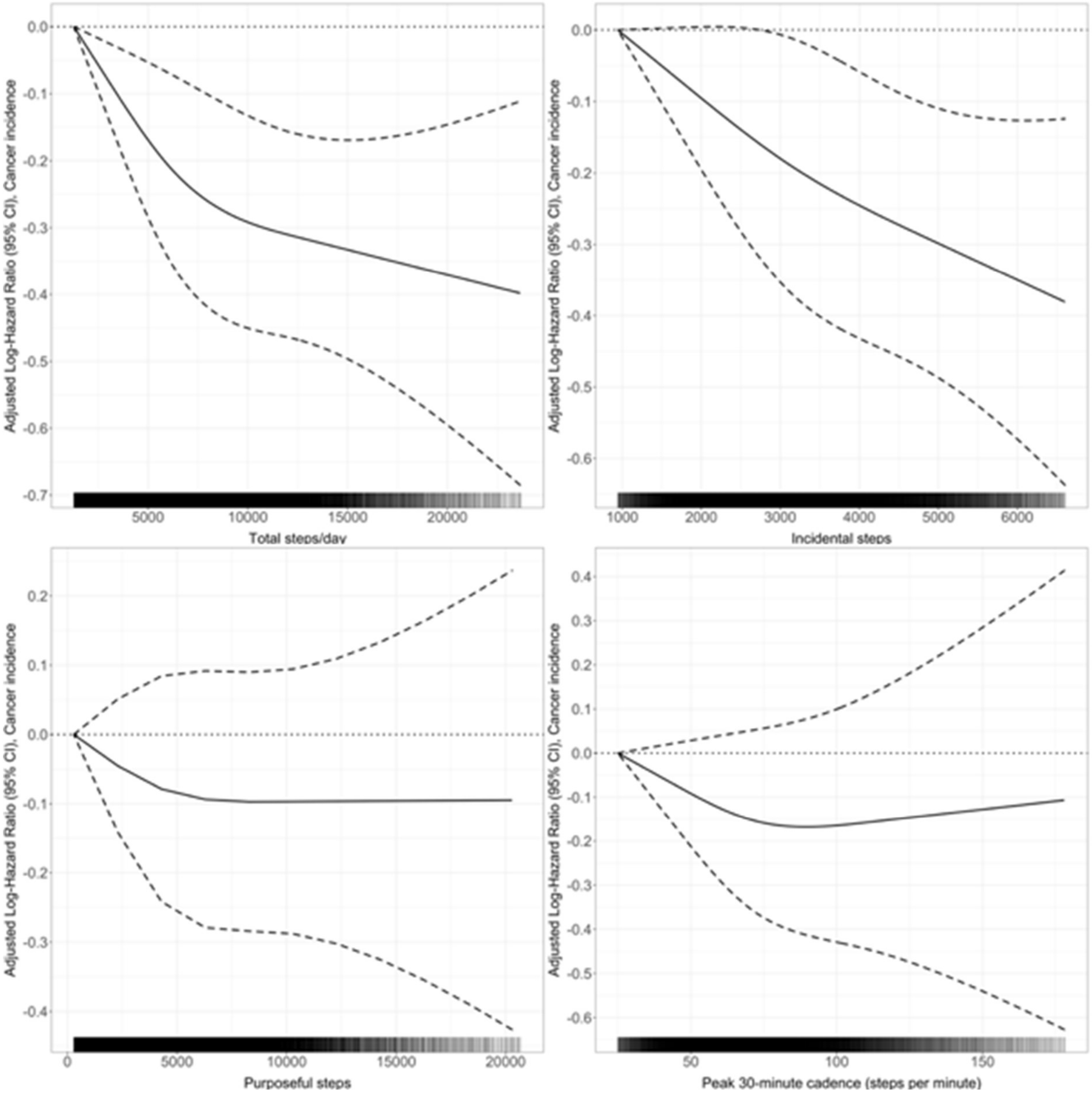

[a]Adjusted for age, sex, ethnicity, education, Townsend deprivation index, smoking, alcohol use, fruit and vegetable consumption, family history of CVD and Cancer, medication use (cholesterol, insulin, and hypertension), accelerometer-measured sleep, and wear accelerometer days. For incidental steps, models were further adjusted for purposeful steps (and vice versa). CVD, Cardiovascular disease; Total steps/day, Average of steps accumulated in a day; Incidental steps, Total daily steps at 1-39 steps/minute; Purposeful steps, Total daily steps at ≥40 steps/minute; Peak 30-min cadence, Average steps/minute recorded for the 30 highest, but not necessarily consecutive, minutes in a day. Dose-response associations were assessed with restricted cubic splines with knots at 10th, 50th, and 90th centiles of the distribution of the exposure of interest. Darker colors in the lower bars represent a higher sample clustering.

© 2022 American Medical Association. All rights reserved.

eFigure 21. Dose–response association (Adjusted[a] hazard ratios and associated 95% confidence interval band) between primary exposures and all-cause mortality (Sensitivity analysis: addition of body mass index, and sleep and dietary score).

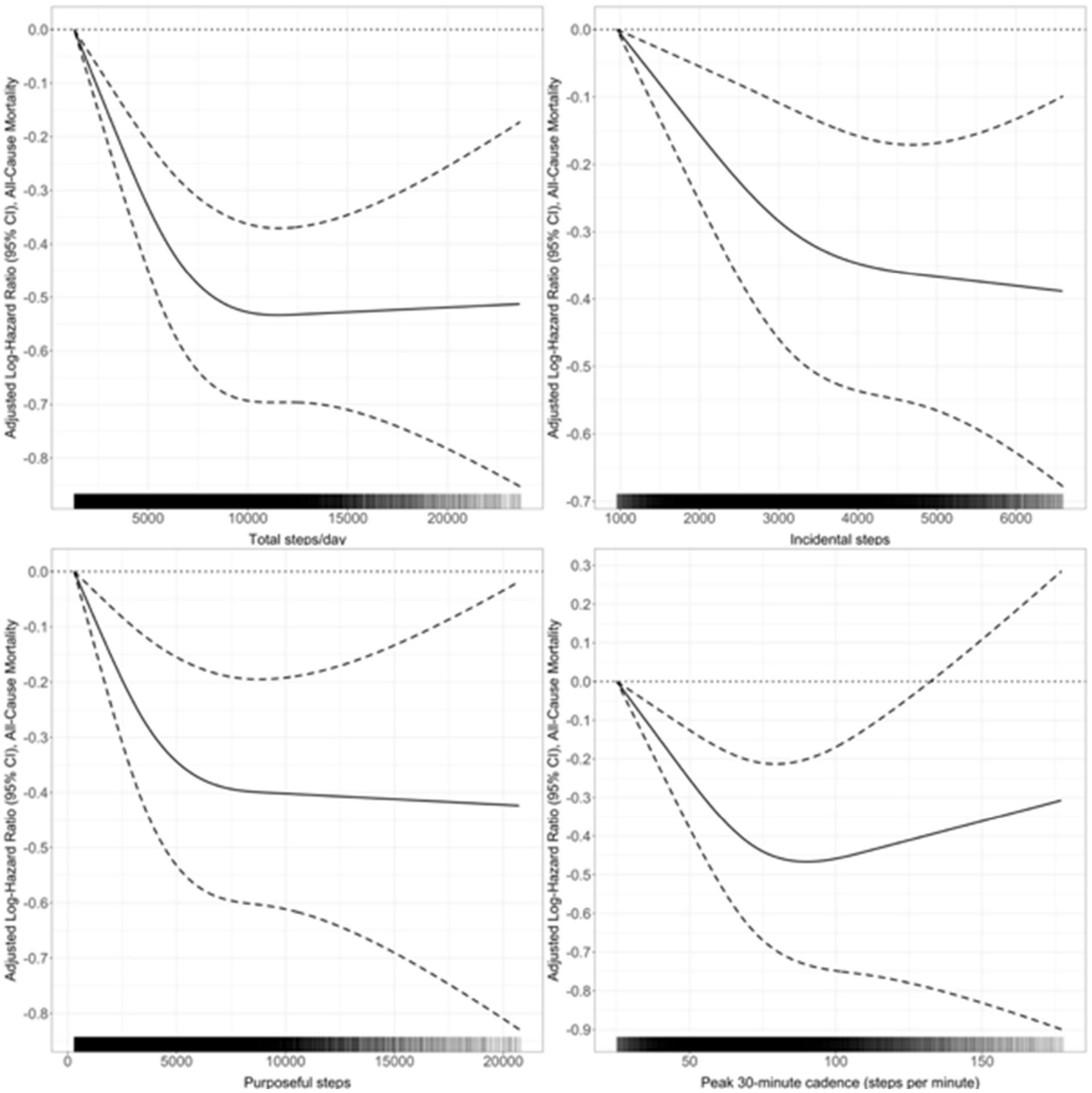

[a]Adjusted for age, sex, ethnicity, education, Townsend deprivation index, smoking, alcohol use, diet quality, family history of CVD and Cancer, medication use (cholesterol, insulin, and hypertension), sleep quality, body mass index, and wear accelerometer days. For incidental steps, models were further adjusted for purposeful steps (and vice versa). CVD, Cardiovascular disease; Total steps/day, Average of steps accumulated in a day; Incidental steps, Total daily steps at 1-39 steps/minute; Purposeful steps, Total daily steps at ≥40 steps/minute; Peak 30-min cadence, Average steps/minute recorded for the 30 highest, but not necessarily consecutive, minutes in a day. Dose-response associations were assessed with restricted cubic splines with knots at 10th, 50th, and 90th centiles of the distribution of the exposure of interest. Darker colors in the lower bars represent a higher sample clustering.

© 2022 American Medical Association. All rights reserved.

eFigure 22. Dose–response association (Adjusted[a] hazard ratios and associated 95% confidence interval band) between primary exposures and CVD mortality (Sensitivity analysis: addition of body mass index, and sleep and dietary score).

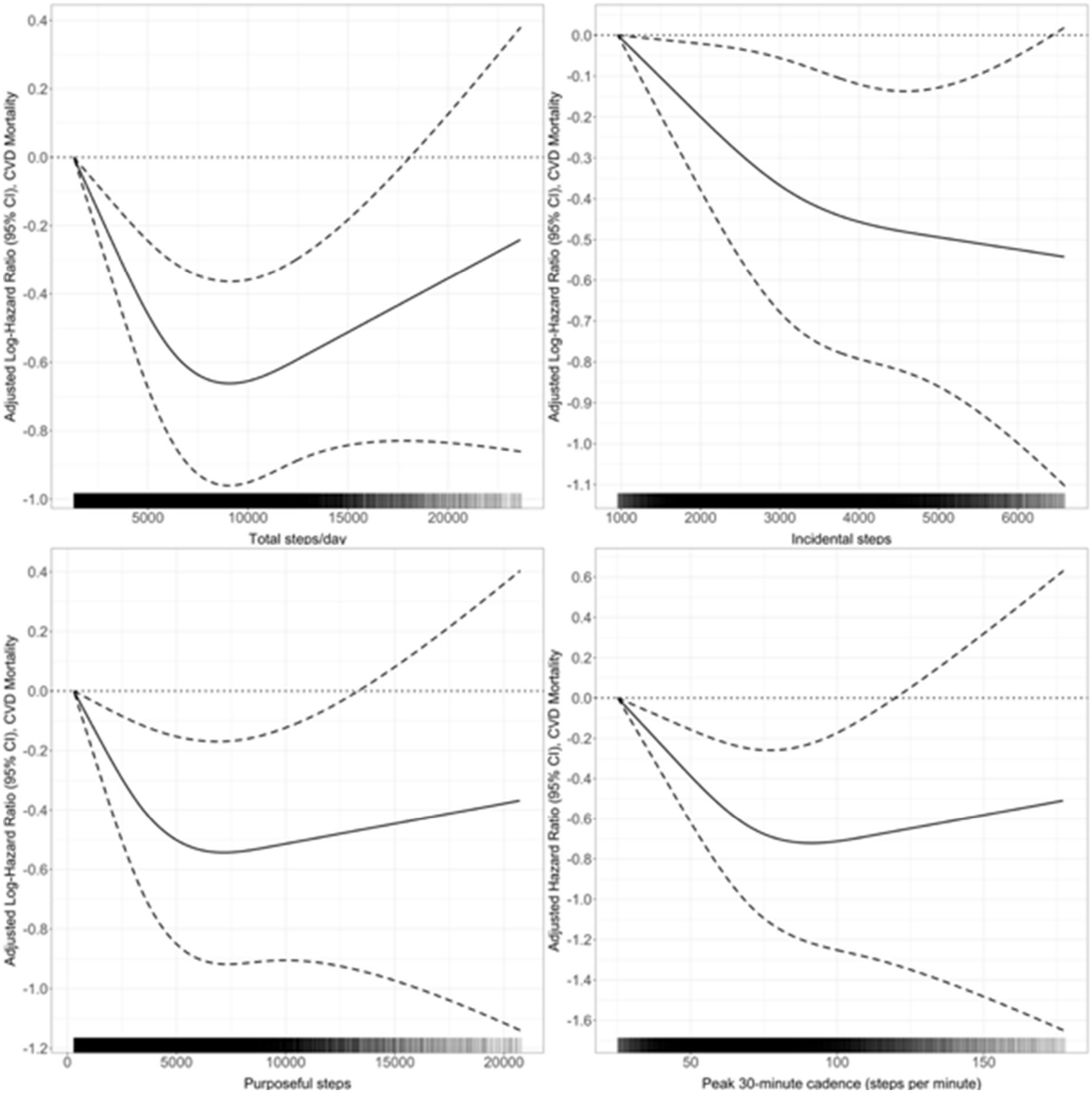

[a]Adjusted for age, sex, ethnicity, education, Townsend deprivation index, smoking, alcohol use, diet quality, family history of CVD and Cancer, medication use (cholesterol, insulin, and hypertension), sleep quality, body mass index, and wear accelerometer days. For incidental steps, models were further adjusted for purposeful steps (and vice versa). CVD, Cardiovascular disease; Total steps/day, Average of steps accumulated in a day; Incidental steps, Total daily steps at 1-39 steps/minute; Purposeful steps, Total daily steps at ≥40 steps/minute; Peak 30-min cadence, Average steps/minute recorded for the 30 highest, but not necessarily consecutive, minutes in a day. Dose-response associations were assessed with restricted cubic splines with knots at 10th, 50th, and 90th centiles of the distribution of the exposure of interest. Darker colors in the lower bars represent a higher sample clustering.

© 2022 American Medical Association. All rights reserved.

eFigure 23. Dose–response association (Adjusted[a] hazard ratios and associated 95% confidence interval band) between primary exposures and physical activity-related cancer composite 13 sites mortality outcome (Sensitivity analysis: addition of body mass index, and sleep and dietary score).

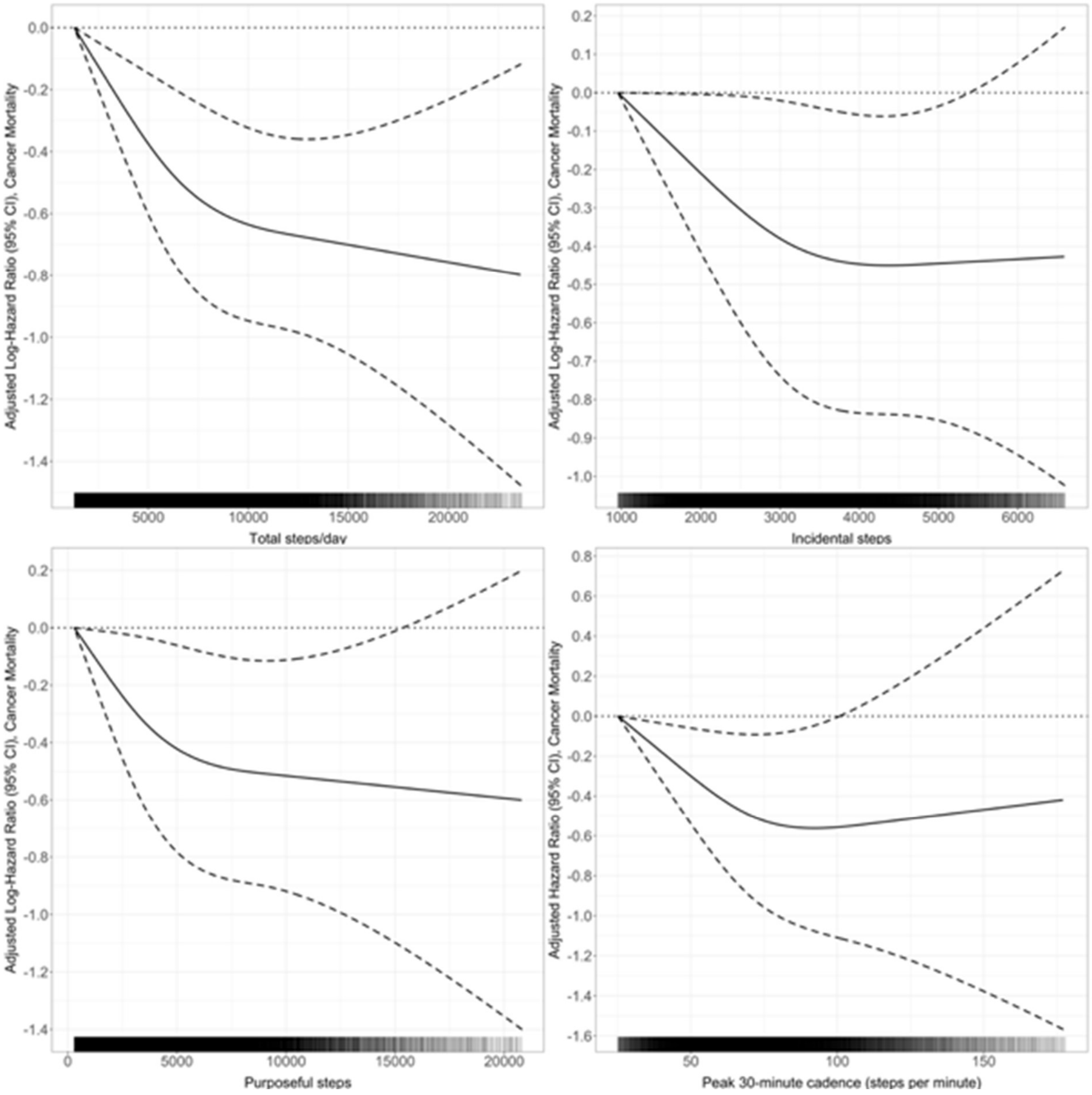

[a]Adjusted for age, sex, ethnicity, education, Townsend deprivation index, smoking, alcohol use, diet quality, family history of CVD and Cancer, medication use (cholesterol, insulin, and hypertension), sleep quality, body mass index, and wear accelerometer days. For incidental steps, models were further adjusted for purposeful steps (and vice versa). CVD, Cardiovascular disease; Total steps/day, Average of steps accumulated in a day; Incidental steps, Total daily steps at 1-39 steps/minute; Purposeful steps, Total daily steps at ≥40 steps/minute; Peak 30-min cadence, Average steps/minute recorded for the 30 highest, but not necessarily consecutive, minutes in a day. Dose-response associations were assessed with restricted cubic splines with knots at 10th, 50th, and 90th centiles of the distribution of the exposure of interest. Darker colors in the lower bars represent a higher sample clustering.

© 2022 American Medical Association. All rights reserved.

eFigure 24. Dose–response association (Adjusted[a] hazard ratios and associated 95% confidence interval band) between primary exposures and CVD incidence (Sensitivity analysis: addition of body mass index, and sleep and dietary score).

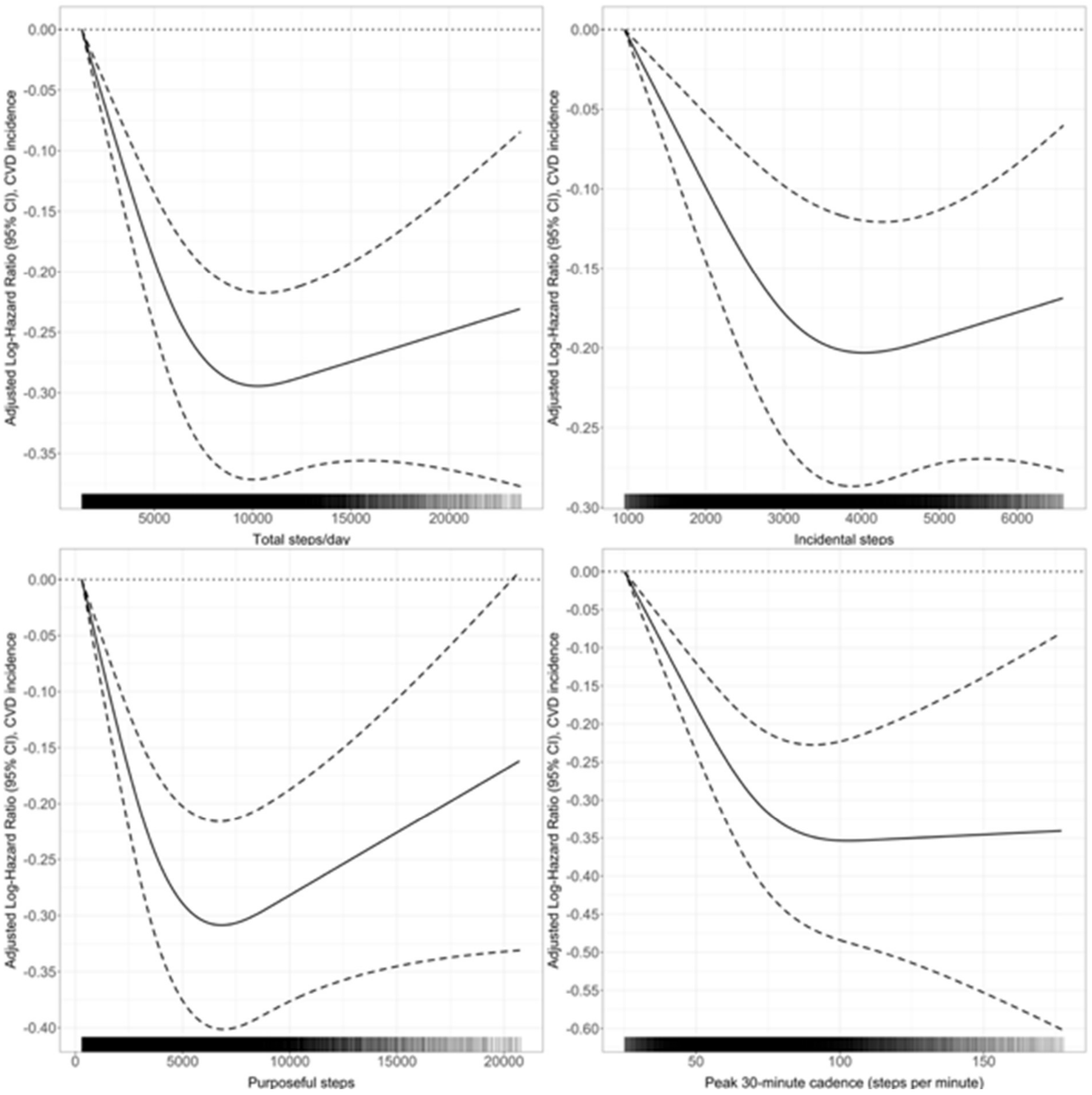

[a]Adjusted for age, sex, ethnicity, education, Townsend deprivation index, smoking, alcohol use, diet quality, family history of CVD and Cancer, medication use (cholesterol, insulin, and hypertension), sleep quality, body mass index, and wear accelerometer days. For incidental steps, models were further adjusted for purposeful steps (and vice versa). CVD, Cardiovascular disease; Total steps/day, Average of steps accumulated in a day; Incidental steps, Total daily steps at 1-39 steps/minute; Purposeful steps, Total daily steps at ≥40 steps/minute; Peak 30-min cadence, Average steps/minute recorded for the 30 highest, but not necessarily consecutive, minutes in a day. Dose-response associations were assessed with restricted cubic splines with knots at 10th, 50th, and 90th centiles of the distribution of the exposure of interest. Darker colors in the lower bars represent a higher sample clustering.

© 2022 American Medical Association. All rights reserved.

eFigure 25. Dose–response association (Adjusted[a] hazard ratios and associated 95% confidence interval band) between primary exposures and physical activity-related cancer composite 13 sites incidence outcome (Sensitivity analysis: addition of body mass index, and sleep and dietary score).

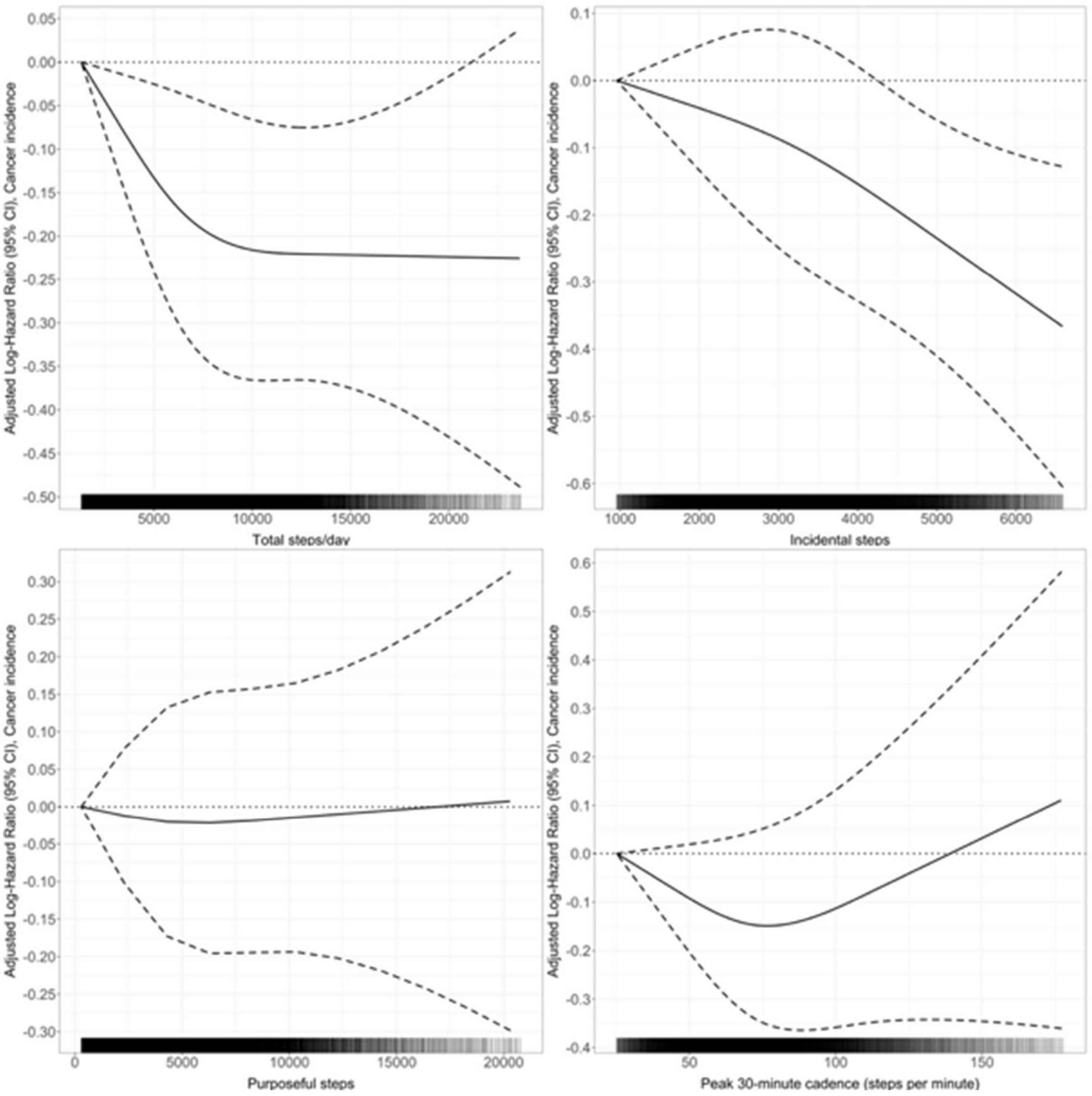

[a]Adjusted for age, sex, ethnicity, education, Townsend deprivation index, smoking, alcohol use, diet quality, family history of CVD and Cancer, medication use (cholesterol, insulin, and hypertension), sleep quality, body mass index, and wear accelerometer days. For incidental steps, models were further adjusted for purposeful steps (and vice versa). CVD, Cardiovascular disease; Total steps/day, Average of steps accumulated in a day; Incidental steps, Total daily steps at 1-39 steps/minute; Purposeful steps, Total daily steps at ≥40 steps/minute; Peak 30-min cadence, Average steps/minute recorded for the 30 highest, but not necessarily consecutive, minutes in a day. Dose-response associations were assessed with restricted cubic splines with knots at 10th, 50th, and 90th centiles of the distribution of the exposure of interest. Darker colors in the lower bars represent a higher sample clustering.

© 2022 American Medical Association. All rights reserved.

eFigure 26. Dose–response association (Adjusted[a] hazard ratios and associated 95% confidence interval band) between primary exposures and all-cause mortality (Sensitivity analysis: restricted to participants with valid wear time days of 20h or more).

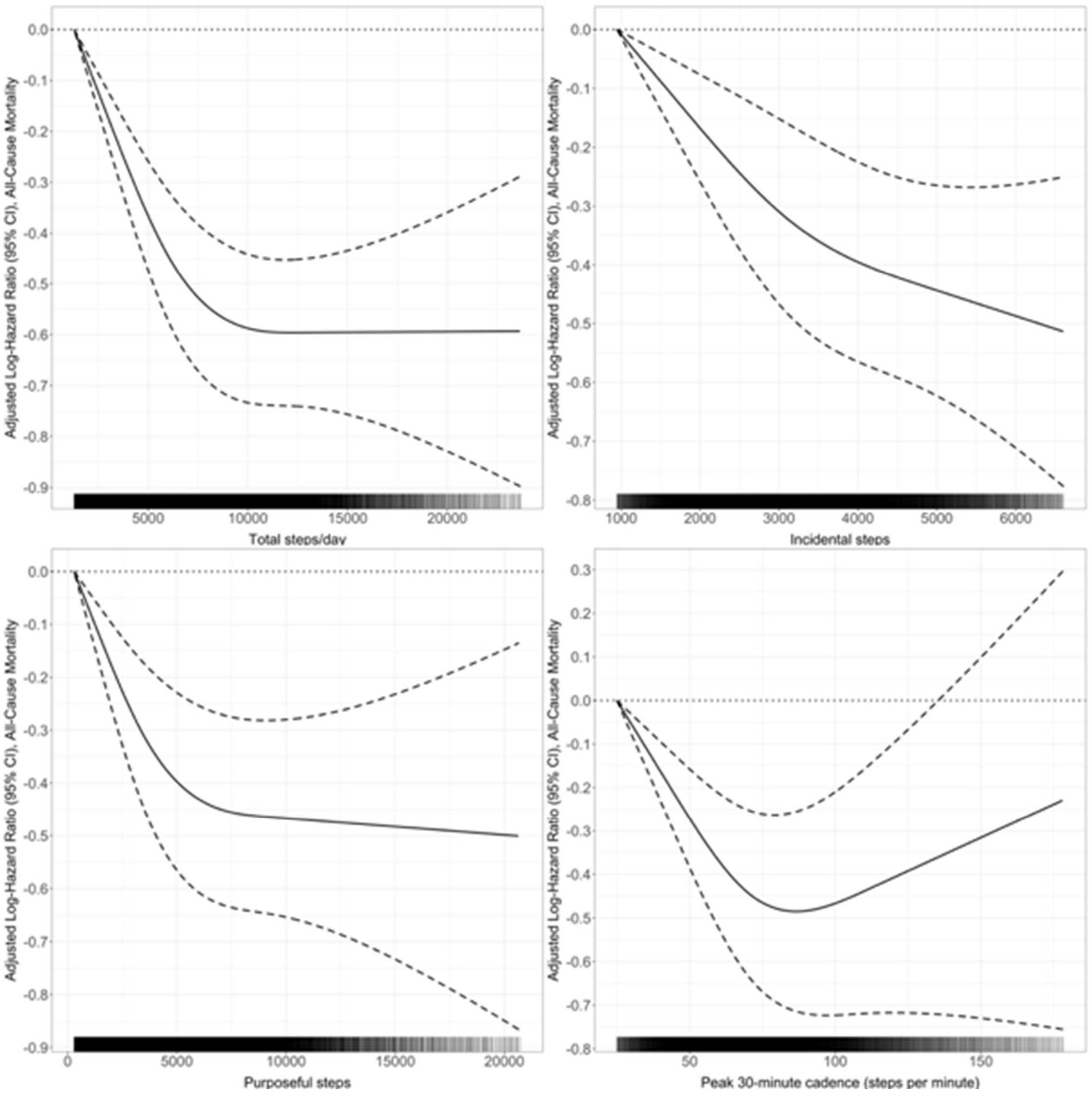

[a]Adjusted for age, sex, ethnicity, education, Townsend deprivation index, smoking, alcohol use, fruit and vegetable consumption, family history of CVD and Cancer, medication use (cholesterol, insulin, and hypertension), accelerometer-measured sleep, and wear accelerometer days. For incidental steps, models were further adjusted for purposeful steps (and vice versa). CVD, Cardiovascular disease; Total steps/day, Average of steps accumulated in a day; Incidental steps, Total daily steps at 1-39 steps/minute; Purposeful steps, Total daily steps at ≥40 steps/minute; Peak 30-min cadence, Average steps/minute recorded for the 30 highest, but not necessarily consecutive, minutes in a day. Dose-response associations were assessed with restricted cubic splines with knots at 10th, 50th, and 90th centiles of the distribution of the exposure of interest. Darker colors in the lower bars represent a higher sample clustering.

© 2022 American Medical Association. All rights reserved.

eFigure 27. Dose–response association (Adjusted[a] hazard ratios and associated 95% confidence interval band) between primary exposures and CVD mortality (Sensitivity analysis: restricted to participants with valid wear time days of 20h or more).

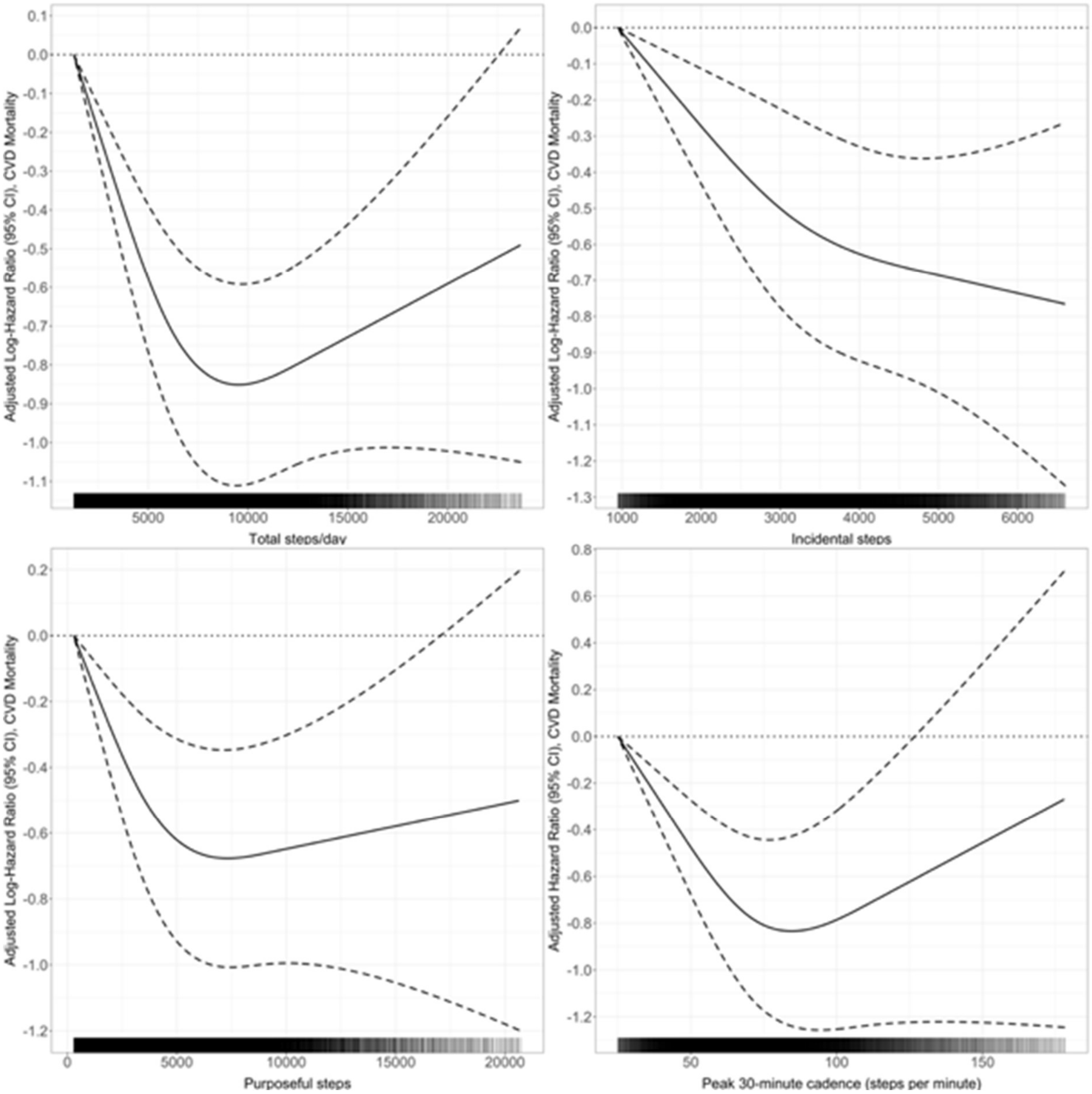

[a]Adjusted for age, sex, ethnicity, education, Townsend deprivation index, smoking, alcohol use, fruit and vegetable consumption, family history of CVD and Cancer, medication use (cholesterol, insulin, and hypertension), accelerometer-measured sleep, and wear accelerometer days. For incidental steps, models were further adjusted for purposeful steps (and vice versa). CVD, Cardiovascular disease; Total steps/day, Average of steps accumulated in a day; Incidental steps, Total daily steps at 1-39 steps/minute; Purposeful steps, Total daily steps at ≥40 steps/minute; Peak 30-min cadence, Average steps/minute recorded for the 30 highest, but not necessarily consecutive, minutes in a day. Dose-response associations were assessed with restricted cubic splines with knots at 10th, 50th, and 90th centiles of the distribution of the exposure of interest. Darker colors in the lower bars represent a higher sample clustering.

© 2022 American Medical Association. All rights reserved.

eFigure 28. Dose–response association (Adjusted[a] hazard ratios and associated 95% confidence interval band) between primary exposures and physical activity-related cancer composite 13 sites mortality outcome (Sensitivity analysis: restricted to participants with valid wear time days of 20h or more).

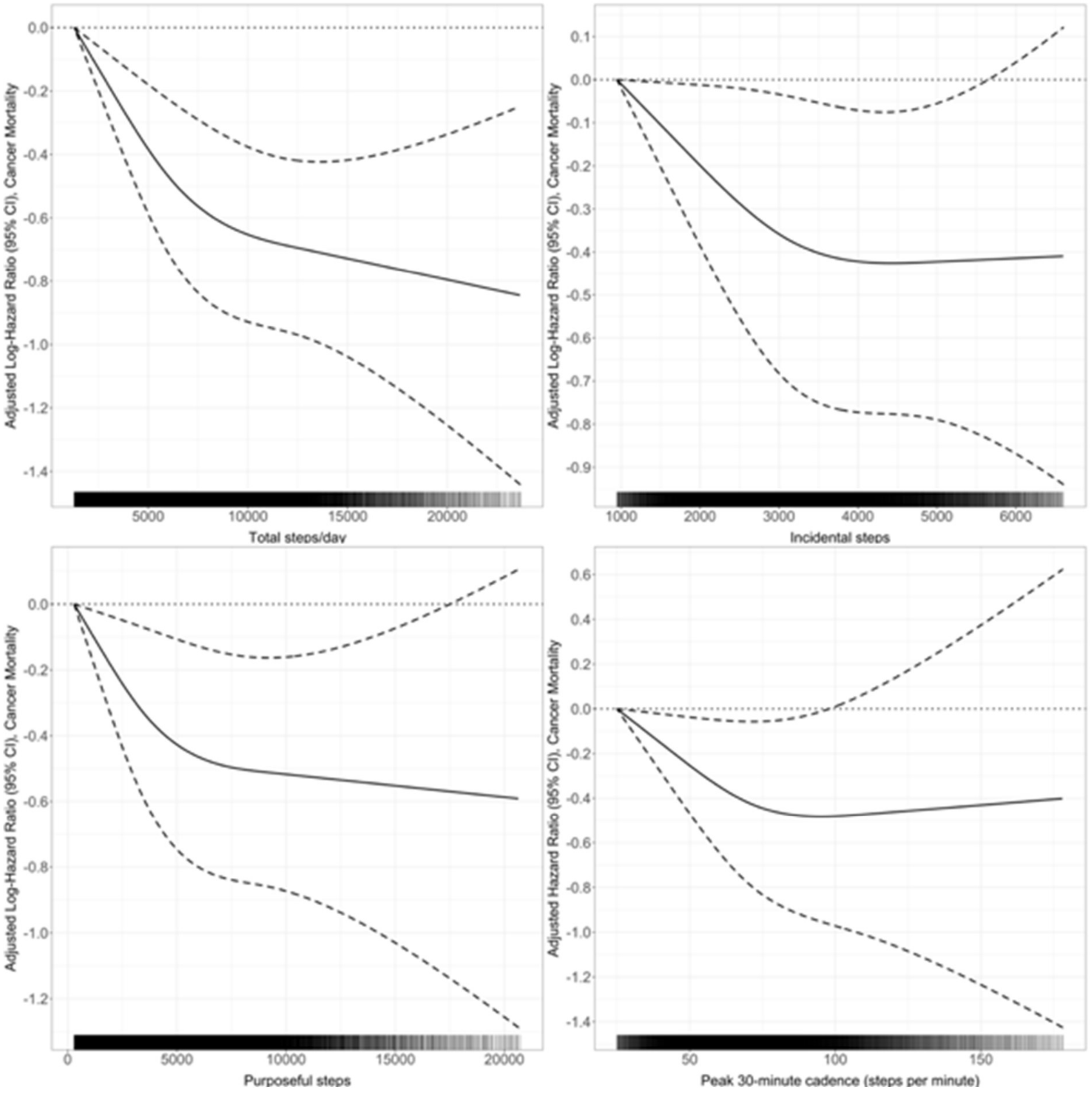

[a]Adjusted for age, sex, ethnicity, education, Townsend deprivation index, smoking, alcohol use, fruit and vegetable consumption, family history of CVD and Cancer, medication use (cholesterol, insulin, and hypertension), accelerometer-measured sleep, and wear accelerometer days. For incidental steps, models were further adjusted for purposeful steps (and vice versa). CVD, Cardiovascular disease; Total steps/day, Average of steps accumulated in a day; Incidental steps, Total daily steps at 1-39 steps/minute; Purposeful steps, Total daily steps at ≥40 steps/minute; Peak 30-min cadence, Average steps/minute recorded for the 30 highest, but not necessarily consecutive, minutes in a day. Dose-response associations were assessed with restricted cubic splines with knots at 10th, 50th, and 90th centiles of the distribution of the exposure of interest. Darker colors in the lower bars represent a higher sample clustering.

© 2022 American Medical Association. All rights reserved.

eFigure 29. Dose–response association (Adjusted[a] hazard ratios and associated 95% confidence interval band) between primary exposures and CVD incidence (Sensitivity analysis: restricted to participants with valid wear time days of 20h or more).

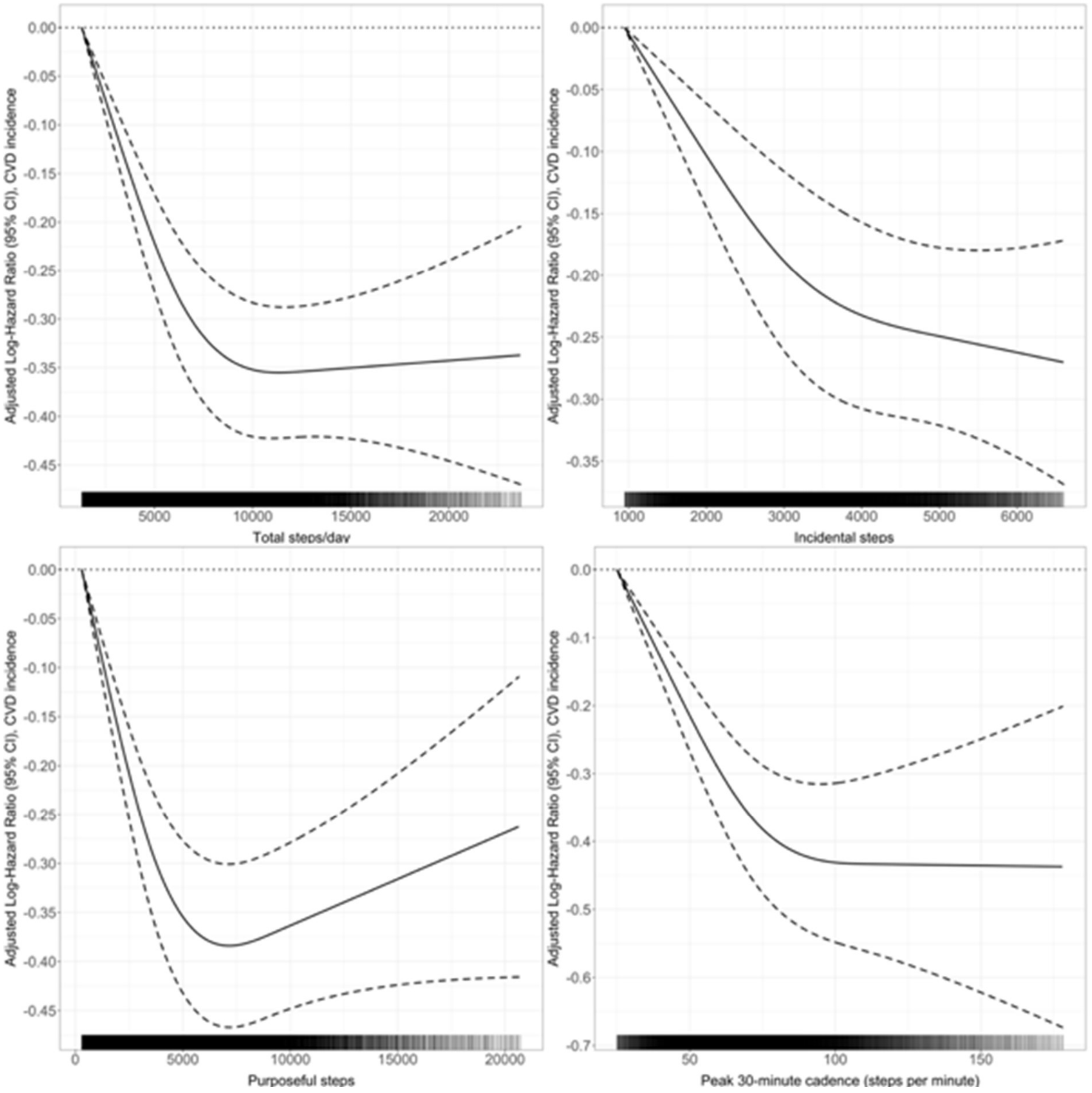

[a]Adjusted for age, sex, ethnicity, education, Townsend deprivation index, smoking, alcohol use, fruit and vegetable consumption, family history of CVD and Cancer, medication use (cholesterol, insulin, and hypertension), accelerometer-measured sleep, and wear accelerometer days. For incidental steps, models were further adjusted for purposeful steps (and vice versa). CVD, Cardiovascular disease; Total steps/day, Average of steps accumulated in a day; Incidental steps, Total daily steps at 1-39 steps/minute; Purposeful steps, Total daily steps at ≥40 steps/minute; Peak 30-min cadence, Average steps/minute recorded for the 30 highest, but not necessarily consecutive, minutes in a day. Dose-response associations were assessed with restricted cubic splines with knots at 10th, 50th, and 90th centiles of the distribution of the exposure of interest. Darker colors in the lower bars represent a higher sample clustering.

© 2022 American Medical Association. All rights reserved.

eFigure 30. Dose–response association (Adjusted[a] hazard ratios and associated 95% confidence interval band) between primary exposures and physical activity-related cancer composite 13 sites incidence outcome (Sensitivity analysis: restricted to participants with valid wear time days of 20h or more).

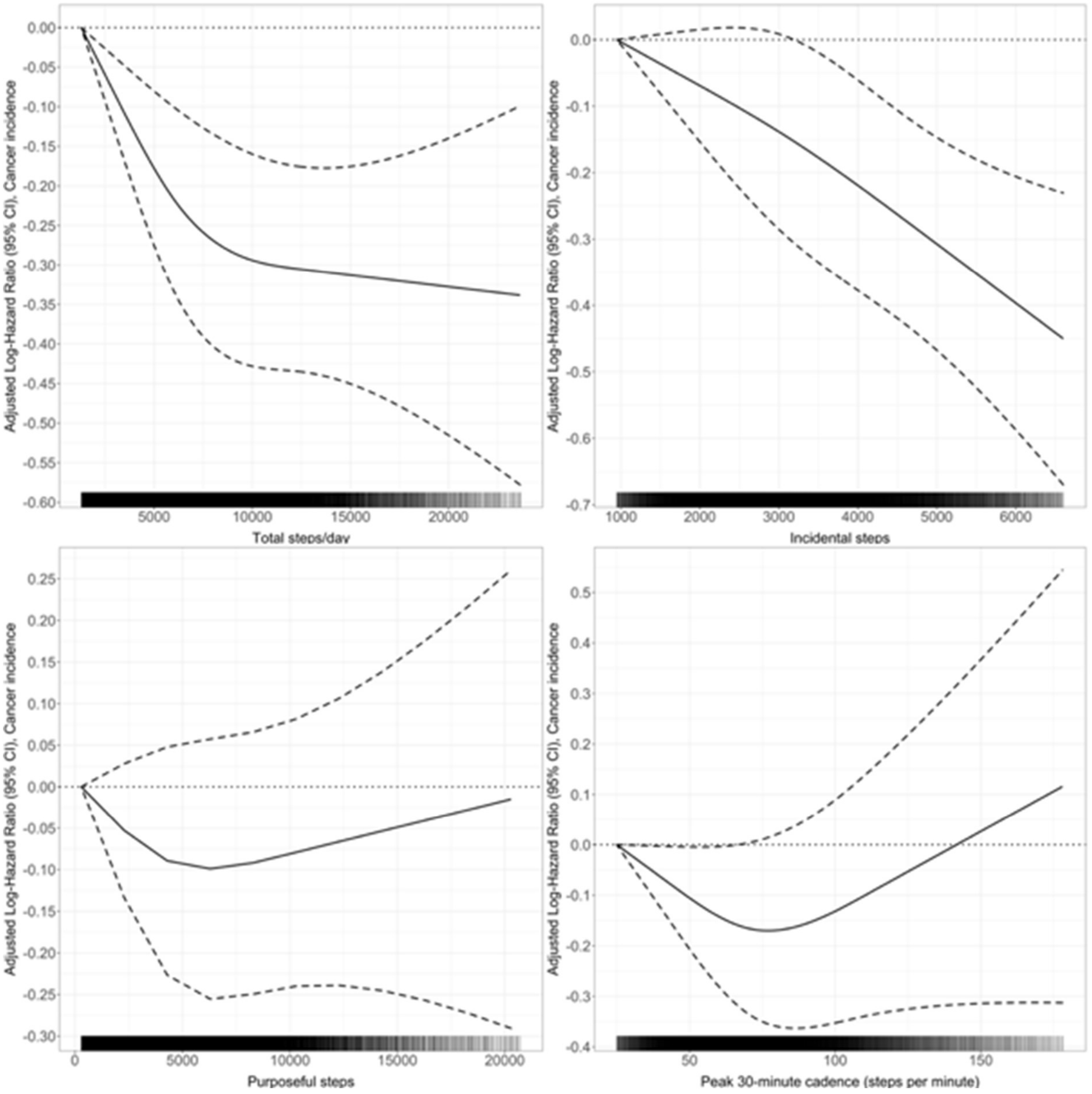

[a]Adjusted for age, sex, ethnicity, education, Townsend deprivation index, smoking, alcohol use, fruit and vegetable consumption, family history of CVD and Cancer, medication use (cholesterol, insulin, and hypertension), accelerometer-measured sleep, and wear accelerometer days. For incidental steps, models were further adjusted for purposeful steps (and vice versa). CVD, Cardiovascular disease; Total steps/day, Average of steps accumulated in a day; Incidental steps, Total daily steps at 1-39 steps/minute; Purposeful steps, Total daily steps at ≥40 steps/minute; Peak 30-min cadence, Average steps/minute recorded for the 30 highest, but not necessarily consecutive, minutes in a day. Dose-response associations were assessed with restricted cubic splines with knots at 10th, 50th, and 90th centiles of the distribution of the exposure of interest. Darker colors in the lower bars represent a higher sample clustering.

© 2022 American Medical Association. All rights reserved.
